# A Super‐Adhesive Air Filter With Capillarity‐Mediated Spontaneous Particle Absorption via Dynamic Bond Exchange

**DOI:** 10.1002/adma.202600006

**Published:** 2026-04-22

**Authors:** Junyong Park, Hyeri Jeon, Hyunwoong Park, Ji Min Lee, Yeomyung Yoon, Gyeong Hwan Choi, Sazzadul A. Rahat, Dong Wook Lee, Sohdam Jeong, Jonathan T. Pham, Chae Bin Kim, Sanghyuk Wooh

**Affiliations:** ^1^ Department of Chemical Engineering Chung‐Ang University Seoul Republic of Korea; ^2^ School of Chemical Engineering Pusan National University Busan Republic of Korea; ^3^ Department of Mechanical and Materials Engineering University of Cincinnati Cincinnati Ohio USA; ^4^ Department of Chemical Engineering Dong‐Eui University Busan Republic of Korea; ^5^ Center for Brain Busan 21 Plus Program Dong‐Eui University Busan Republic of Korea; ^6^ Department of Chemical and Environmental Engineering University of Cincinnati Cincinnati Ohio USA; ^7^ Department of Polymer Science and Engineering Pusan National University Busan Republic of Korea; ^8^ Research Institute of Industrial Technology Pusan National University Busan Republic of Korea; ^9^ Research Institute for Convergence of Biomedical Science and Technology Pusan National University Yangsan Hospital Yangsan Republic of Korea

**Keywords:** air filter, capillary force, dynamic bond polymer, particle adhesion, viscoelasticity

## Abstract

A strategy to coat a thin liquid film over substrates enables strong particle adhesion via capillary force. However, the practical utility of a liquid film is hindered by inherent fluid instabilities, including the Plateau‐Rayleigh instability, interfacial dewetting, and drainage. Here, we introduce dynamically crosslinked adhesive films that maintain an elastic network to ensure structural integrity while enabling viscous, capillarity‐driven particle adhesion. Polydimethylsiloxane with an imine crosslinked network is applied as a viscoelastic adhesive. While the elastic nature allows for stable and thick films, it remains sufficiently viscous to form a meniscus upon particle contact; this generates a micro‐Newton scale, strong capillary force. When applied to air filters, which typically rely on nano‐Newton scale van der Waals adhesion, the dynamically crosslinked adhesive improves filtration performance and suppresses particle resuspension. Rather than only retaining particles on the surface, the adhesive layer with dynamic imine bonds actively absorbs particles into the matrix, which then exposes a fresh sticky surface for sustained filtering and prolongs the increase in pressure drop by filtering. Therefore, operational lifetime of the filter is extended compared to conventional filters, even with more effective filtration. These unique particle capturing characteristics of a stable dynamic bond adhesive layer not only exhibits outstanding filtration performances, but also proposes a new paradigm of air purification technologies.

## Graphical abstract

We address the inherently weak particle adhesion of conventional air filters by coating a dynamically crosslinked adhesive layer that delivers capillarity‐driven strong adhesion and particle absorption mediated by dynamic bond exchange. The resulting enhancement in particle adhesion enables efficient ultrafast (up to 20 m s^−1^) and omnidirectional filtration, while particle absorption mitigates clogging and substantially extends filter lifetime.

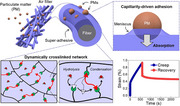

## 1 Introduction

Particulate matter (PM) capture on solid surfaces relies mainly on particle adhesion via van der Waals interaction [1, 2, 3]. Due to the nano‐Newton scale weak adhesion, captured PMs are easily bounced off and/or resuspended [4, 5]. In fibrous air filtration systems, airborne particles are intercepted by fibers and must remain adhered to the surface to ensure effective removal of PMs from the airstream. Therefore, insufficient adhesion directly limits filtration performance and may result in secondary contamination. Though reducing pore size can improve filtration efficiency, at the same time, a higher pressure drop is unavoidably produced. High initial pressure drop inevitably increases the power consumption. In recent years, filter design has focused on increasing the surface area of the internal filter media by using nanofiber media [6, 7, 8]. This approach improves filtration efficiency with a smaller pressure drop increase; however, the long‐standing issue of weak particle adhesion still remains for PMs filtration.

When a thin liquid film is introduced onto a solid surface, a liquid meniscus forms at the particle surface and generates micro‐Newton scale capillary forces that greatly exceed van der Waals interactions [9, 10]. Inspired by this principle, a thin liquid film coating on air filters, which biomimics the mucus layer of human nasal hairs, has emerged as a promising strategy to overcome this adhesion limit [11]. The liquid layer exerts strong capillary forces on captured PMs, significantly enhancing filtering performance; this filter methodology is named the particle removing oil‐coated (PRO) filter. In contrast to conventional filters, the PRO filter prevents detachment of captured PM and enables efficient filtration under fast airflow. Furthermore, the capillary‐driven packing of captured PMs effectively delays the pressure drop increase. Although previous efforts attempted to stabilize this thin liquid layer through surface modification, it remains mechanically vulnerable due to its intrinsic fluidity. Moreover, when the liquid layer becomes thicker, the film breaks up into droplets on the fiber surface due to a Plateau‐Rayleigh instability driven by surface tension [12, 13, 14]. Such surface droplets subsequently obstruct the filter pores and increase the pressure drop across the filter media [15, 16, 17]. In addition, the liquid layer can experience sufficient drag forces to detach under airflow, and the resulting airborne droplets can lead to secondary contamination [18, 19].

**FIGURE 1:**
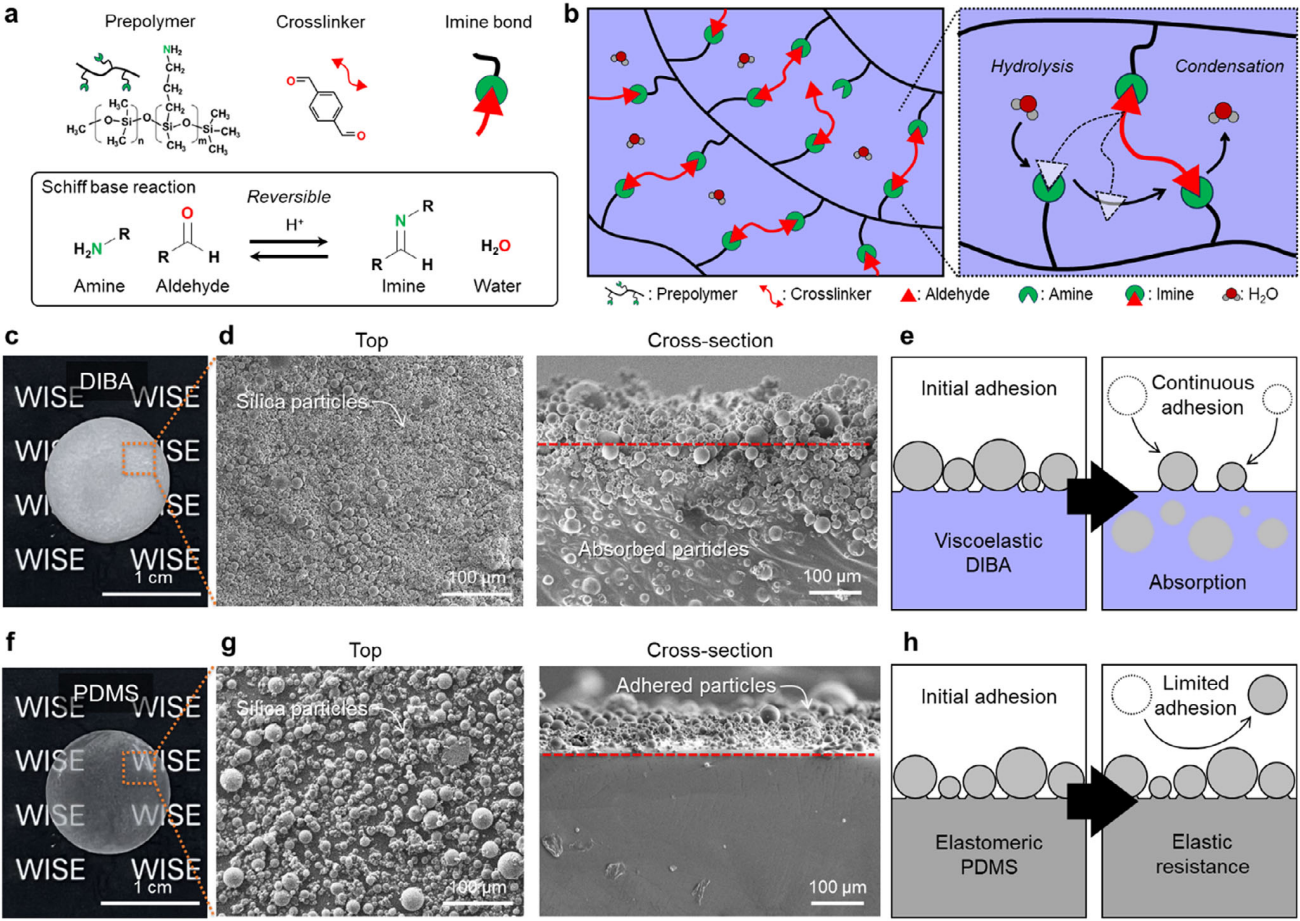
Capillarity‐driven particulate‐absorbing adhesive. (a) Schematic illustrating prepolymer, crosslinker and resulting imine bond (top) based on Shiff base reaction (bottom). (b) Schematics of crosslink network with covalent imine bond (left) and bond exchange mediated by inherent water molecule (right). The dynamic network consists of prepolymers (green circles with black lines), crosslinker (red wavy arrows), and water molecules. (c,f) Photographs of (c) DIBA (∼550 µm) and (f) permanently crosslinked PDMS film (∼560 µm) covered with silica microparticle (5–50 µm). (d,g) The corresponding top‐view (left) and cross‐sectional (right) scanning electron microscope (SEM) images of (d) DIBA and (g) PDMS. The orange and red dotted line indicated zoomed area and top edge of the samples, respectively. (e,h) Schematics of (e) continuous particle adhesion and absorption of viscoelastic DIBA with dynamic bond exchange and (h) limited particle adhesion for elastomeric PDMS with permanently crosslinked network.

Crosslinking polymeric chains is one of the most effective approaches to enhance mechanical stability by forming a continuous network [20]. However, the crosslinked network suppresses molecular mobility, and the resulting elasticity hinders the formation of a meniscus on particles [21]. Therefore, even when stability is achieved via crosslinking, some flowability must be retained to form a sufficiently large meniscus and sustain capillary adhesion on PMs. Polymers with covalent adaptable networks (CANs), capable of reversible bond exchange, are attracting broad interest due to their combination of elastic stability and viscous flow through network rearrangement [22, 23]. Although the network is covalently crosslinked, the dynamic bond exchange allows the network to behave as a viscoelastic fluid with tunable, long‐term relaxation. Thus, the CANs have been applied in a wide range of smart materials including recyclable thermoset polymers [24, 25, 26, 27], self‐healing systems [28, 29], shape‐morphing polymers [30, 31, 32, 33], soft robotics [34], and 3D printing resins [35]. Especially, the CANs offer a distinctive advantage as adhesives by leveraging reversible dynamic bonds [36, 37]. Their crosslinked network provides mechanical durability, while their flow behavior through dynamic bond exchange enables the adhesive to effectively wet the surface [38]. Unique viscoelastic properties of adaptable networks can allow the adhesive to develop a particle‐bound meniscus capable of producing strong capillary adhesion. Representative dynamic bonds include imine bond [39, 40, 41], boronate ester bond [42, 43], disulfide exchange [44, 45], transesterification [46, 47], metal‐ligand coordination bond [48, 49], and Diels‐Alder linkages [50, 51]. Dynamic bond exchanges are broadly classified into associative and dissociative types. The associative bond exchange proceeds through a higher‐coordinate intermediate in which a new bond forms before the original bond breaks. In contrast, the dissociative mechanism involves initial bond cleavage that regenerates the reactants and forms a new bond. Among dynamic covalent chemistries, imine bonds can undergo both associative and dissociative reversible bond exchange. Imine metathesis proceeds via an associative mechanism at elevated temperature, and is generally employed for a bond exchange reaction [40, 41]. However, imine bonds are capable of dynamic exchange via a dissociative mechanism even at room temperature through Schiff base chemistry [40, 52, 53]. Since imine bonding follows reversible equilibrium, based on the Schiff base reaction depicted in Figure 1a, water enables continuous cycles of hydrolysis and condensation of the network at ambient conditions [52]. Given that, an imine‐crosslinked network can maintain viscoelastic behavior even under typical filtration conditions that imparts liquid‐like capillary‐driven adhesion, because water‐mediated bond exchange remains active at room temperature (25°C).

Here, we employed an imine‐based, dynamically crosslinked network as a mechanically stable adhesive layer for robust PM capture; we named it the dynamic imine bond adhesive (DIBA). The DIBA demonstrated spontaneous bond exchange under ambient conditions and its viscoelastic behavior was confirmed through rheological characterization. The dynamic bond exchange enables the DIBA to form a meniscus around particles and to provide strong capillary adhesion comparable to liquid coatings, while DIBA remains mechanically robust under external physical abrasion and ultrafast airflow. The elasticity of DIBA prevents both the Plateau‐Rayleigh instability and the detachment of particles under airflow. As a result, these dynamic super‐adhesive filters exhibited significantly enhanced filtration performance. Importantly, the DIBA layer increased filtering efficiency without altering the pressure drop and maintained its filtering performance even under ultrafast face velocity (up to 20 m s^−1^). Furthermore, viscoelastic flow allowed surface‐adhered PMs to be absorbed into the crosslinked network, similar to pure low viscosity oils [11]. Such absorption‐driven densification suppresses pore clogging and significantly extends the operational lifetime of the filter.

## 2 Results and Discussion

### 2.1 General Approach

We synthesized a DIBA through Schiff base condensation by mixing amine‐modified polydimethylsiloxane (AmPDMS) and terephthalaldehyde, acting as prepolymer and crosslinker, respectively (Figure 1a) [54]. Water is generated as a byproduct of the crosslinking reaction, remaining within the network. The reversible reaction proceeds in dynamic equilibrium characterized by its equilibrium constants (*K_eq_*) (Figure 1b) [40]. Conventional imine‐based polymers have typically been designed to remove water molecules after synthesis to maximize the yield of bond formation. In this study, however, the remaining water molecules serve as mediators to facilitate hydrolysis of imine (Figure 1b, right). The resulting amine and aldehyde groups subsequently recombine, which enables dissociative bond rearrangement within the crosslinked network.

Owing to the viscoelastic flow enabled by dynamic bond exchange, DIBA exhibits strong adhesion to particles and can progressively absorb captured particles into the bulk matrix. To characterize the particle adhesion and absorption behavior, we imaged DIBA and permanently crosslinked polydimethylsiloxane (PDMS without dynamic bond characteristics) after the silica particles (5–50 µm) were manually applied through a gentle sieving. Both DIBA and PDMS samples were prepared by drop‐casting onto glass substrates with resulting thicknesses of 550.8 ± 86.9 and 562.6 ± 63.9 µm, respectively. The PDMS elastomer was fabricated using the Sylgard 184 kit, and its base‐crosslinker ratio was adjusted to match the storage modulus (G′) of DIBA (Figure S1). Top‐view photographs of the samples revealed a clear contrast; while the initially transparent samples turned opaque upon particle capturing, the DIBA sample appeared more opaque than the PDMS due to absorbed silica particles (Figure 1c,f, Figure S1). Scanning electron microscope (SEM) top‐view images further show this difference that the DIBA surface was uniformly covered with densely packed silica particles (Figure 1d, left), whereas PDMS demonstrated patchy, discontinuous particle adhesion (Figure 1g, left). Cross‐sectional SEM imaging further showed that silica particles were embedded within the DIBA matrix (Figure 1d, right). Penetration of adhered particles exposes newly generated adhesive surfaces around the particle, enabling continued capture of PMs with strong adhesion (Figure 1e). In contrast, the PDMS sample demonstrated only surface‐adhered particles in the cross‐section SEM image (Figure 1g, right). Since its elastic network resists deformation and prevents further penetration, PDMS samples exhibited surface‐limited adhesion (Figure 1h). This demonstrated that DIBA provided both strong adhesion and progressive absorption into the matrix. Such particle‐capture behavior is expected to make DIBA a highly effective adhesive layer for air filtration.

### 2.2 Rheological Properties of DIBA

**FIGURE 2:**
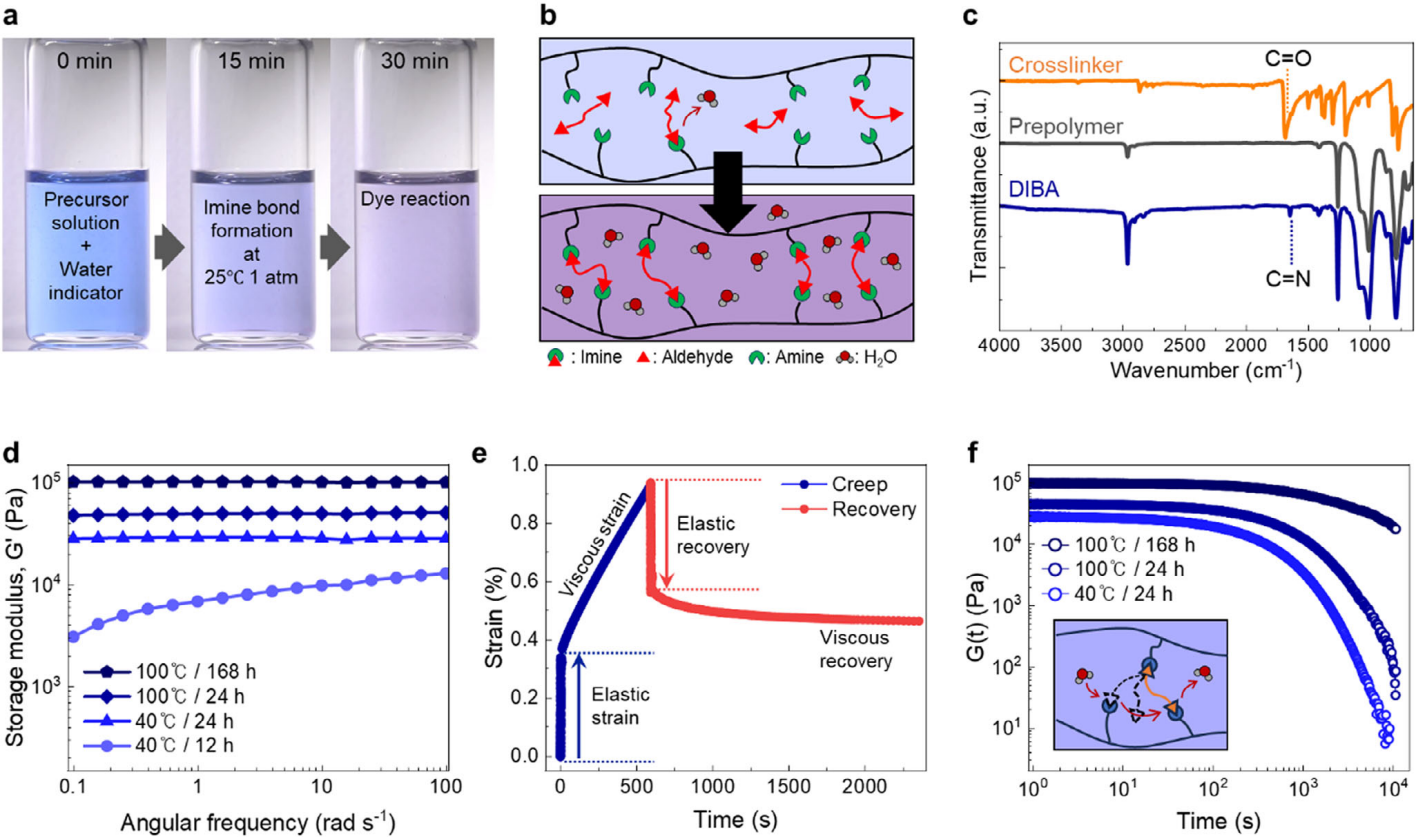
Rheological property of DIBA. (a) Snapshots of the reaction of the DIBA prepolymer and crosslinker solution mixed with water indicator (CoCl_2_) under 25°C at 1 atm inside a sealed reaction chamber isolated from the external environment. (b) Schematic illustrating imine crosslinking reaction and the produced water molecules that are changing the color of network. (c) Fourier‐transform infrared (FTIR) spectra of the crosslinker (orange), prepolymer (gray), and crosslinked DIBA (blue). (d) Frequency sweep of DIBA under varied post‐drying conditions at 1% strain from 0.1 to 100 rad s^−1^. (e) Creep (blue line)‐recovery (red line) of DIBA post‐dried under vacuum at 40°C for 24 h for applying 100 Pa of shear stress. (f) Stress relaxation curves as a function of post‐drying conditions at 1% strain at 25°C. All rheological measurement was operated under 25°C. Inner schematic illustrates bond rearrangement mediated by water molecule that facilitates DIBA to relieve stress without external stimuli.

The synthesis of DIBA is a simple process involving (1) mixing the prepolymer and crosslinker solutions, and (2) drying to remove the solvent and to adjust the water level to achieve a stable crosslinked network. Notably, the polymerization of imine bonds spontaneously proceeds at room temperature (25°C). We visually demonstrated that imine bond formation occurs within the precursor solution composed of the prepolymer and crosslinker solution and its resulting water using anhydrous cobalt(II) chloride (CoCl_2_), a water indicator (Figure 2a). To observe only the water molecules generated by imine bond formation, the precursor solution was isolated from the external environment during the reaction. The changes in solution color indicated that anhydrous CoCl_2_ (blue) coordinated with the water molecules generated during the Schiff base condensation, forming hydrated hexaaquiacobalt(II) complex, Co(H_2_O)_6_^2+^ (pink) (Figure 2b). The crosslinking reaction of DIBA proceeds spontaneously without external energy input, and the generated water molecules remain entrapped within the network. Initial imine formation under anhydrous and neutral conditions of precursor solution is driven by a proton‐transfer mechanism, where amine groups on the prepolymer act as proton donors to activate carbonyl groups of neighboring aldehydes [55]. The formation of imine bonds was confirmed by Fourier‐transform infrared (FTIR) spectroscopy of the reactants and resulting polymer (Figure 2c). The characteristic C═O stretching vibration of terephthalaldehyde was absorbed near ∼1700 cm^−1^. Notably, a new absorption band appeared at ∼1650 cm^−1^, corresponding to the characteristic C═N stretching vibration of the imine bond. Spectral changes provide direct evidence that imine bonds were successfully formed between the prepolymer and the crosslinker. Differential scanning calorimetry (DSC) measurement performed from −90°C to 150°C did not reveal a discernible thermal transition (Figure S2). This may be attributed to the intrinsically low glass transition temperature of PDMS‐based imine networks, which has been reported to occur near −120°C, confirming that DIBA remains in a rubbery state under ambient conditions [54]. Thermogravimetric analysis (TGA) showed no significant mass loss prior to thermal degradation above ∼400°C, indicating the absence of residual solvent and good thermal stability of the crosslinked network (Figure S2).

Since the imine bond formation is a reversible reaction in equilibrium, the water content within the matrix is a key factor that determines the mechanical properties of DIBA. We fabricated DIBA by mixing prepolymer with crosslinker in 1:0.75 molar ratio and allowing the solvent to evaporate at 25°C under ambient pressure. The internal water content was then adjusted through post‐drying at 40°C or 100°C under vacuum. The corresponding changes in viscoelastic behavior were analyzed using a rheometer (Figure 2d). All rheological experiments were performed using a 1% strain condition, which is within the linear viscoelastic region (Figure S3). The sample post‐dried in vacuum at 40°C for 12 h exhibited a frequency dependence of the storage modulus (G′), within the frequency range measured. Such behavior is characteristic of a viscoelastic liquid arising from chain relaxation of uncrosslinked prepolymers [56]. However, when the drying time was extended to 24 h, G′ began to exhibit a frequency‐independent plateau region. This confirmed that a stable crosslinked network was formed when internal water molecules were sufficiently removed, at least within the range of measured frequency. Furthermore, when the water was further removed by drying under vacuum at 100°C for 24 or 168 h, a substantial increase in G′ was observed, verifying that lower water content leads to stiffer material.

Furthermore, mechanical properties of DIBA can also be adjusted by modifying the mixing ratio of the prepolymer and the crosslinker (Figure S4). The amine‐functionalized prepolymer contains approximately four amine groups per chain, while the crosslinker provides two aldehyde groups per molecules; therefore, a molar mixing ratio of prepolymer to crosslinker of 1:2 corresponds to an approximately 1:1 stoichiometric equivalence of reactive amine and aldehyde functionalities. We analyzed the viscoelastic properties while varying the mixing ratio of samples dried under vacuum at 40°C for 24 h, which ensured the formation of a stable network. We confirmed that the frequency‐independent plateau in G′ was achieved only when the prepolymer to crosslinker molar ratio reached 1:0.75. In this case, DIBA is characterized as a viscoelastic solid within this frequency range, as its storage modulus (G′) exceeds its loss modulus (G″). In contrast, DIBA with a lower crosslinker ratio, such as 1:0.5, showed liquid characteristic (G″ > G′). The difference in rheological behavior was further evident from a macroscopic mechanical contact test (Figure S5). The 1:0.75 DIBA left no residue on the probe surface. However, the 1:0.5 DIBA left a sticky residue, similar to that of a viscous liquid oil. Therefore, subsequent experiments were conducted using a mixing ratio of 1:0.75, which is the minimum ratio required to form a mechanically stable crosslinked matrix.

Although DIBA forms a crosslinked network that provides elastic structural integrity, the rearrangement of reversible bonds triggered by water imparts viscous behavior that plays a crucial role in particle capturing. In other words, DIBA behaves as a solid within the 0.1 to 100 rad s^−1^ frequency range, but will still fully relax like a liquid at longer time scales. To characterize the creep behavior of DIBA, we conducted a typical creep‐recovery test (Figure 2e). When a constant shear stress of 100 Pa was applied, DIBA exhibited instantaneous strain, characteristic of elastic behavior. The strain gradually increased over time through creep. Subsequently, the stress is removed, and an instantaneous recovery of nearly the same magnitude as the initial elastic strain was observed. The following strain slowly decreased due to a delayed viscoelastic flow recovery, which can be attributed to relaxation enabled by the reversible imine bonds. The creep‐recovery results demonstrate that the DIBA possesses both the solid‐like and liquid‐like characteristics. In addition to creep, the continuous local dissociation and reformation of covalent bonds allows topological rearrangement of polymer chains, enabling DIBA to relax without external energy input. This is confirmed through a stress relaxation measurement with a constant 1% strain at 25°C (Figure 2f). With increased crosslink density, a longer relaxation time required. Even after extreme drying at 100°C for 7 days, stress relaxation was still observed, but with a prolonged relaxation time. Stress relaxation of DIBA at 25°C without external stimuli suggests that residual water remains strongly bound in the network and is sufficient to facilitate bond exchange.

To further validate the unique viscoelastic behavior of DIBA and its water‐dependent stress relaxation mechanism by dissociative bond exchange, we performed nonequilibrium molecular dynamics (NEMD) simulations (Figure S6). An atomistic model of the DIBA network was first constructed. Stress relaxation was then evaluated as a function of the crosslinking ratio (which is similar to the experimentally controlled drying duration) after imposing a uniaxial strain, based on the probability of the dynamic bond exchange reaction computed from density functional theory (DFT) calculations. The simulation results reveal that the imine formation increases by removing the water content in the system, resulting in a higher effective crosslink density (from crosslinking 50% to 100%). We confirmed that the increase in crosslink density leads to a relatively slower stress relaxation (Figure S6b). The simulated stress relaxation results validated the experimental observation that prolonged drying times led to longer stress relaxation. Additionally, the NEMD results show water molecules induce local bond exchange by catalyzing the hydrolysis and subsequent reformation of imine bonds, even under ambient conditions (Figure S6c). Such atomic‐level rearrangement effectively dissipated the applied stress, leading to macroscopic stress relaxation.

### 2.3 Capillary Adhesion of Particle on DIBA

**FIGURE 3:**
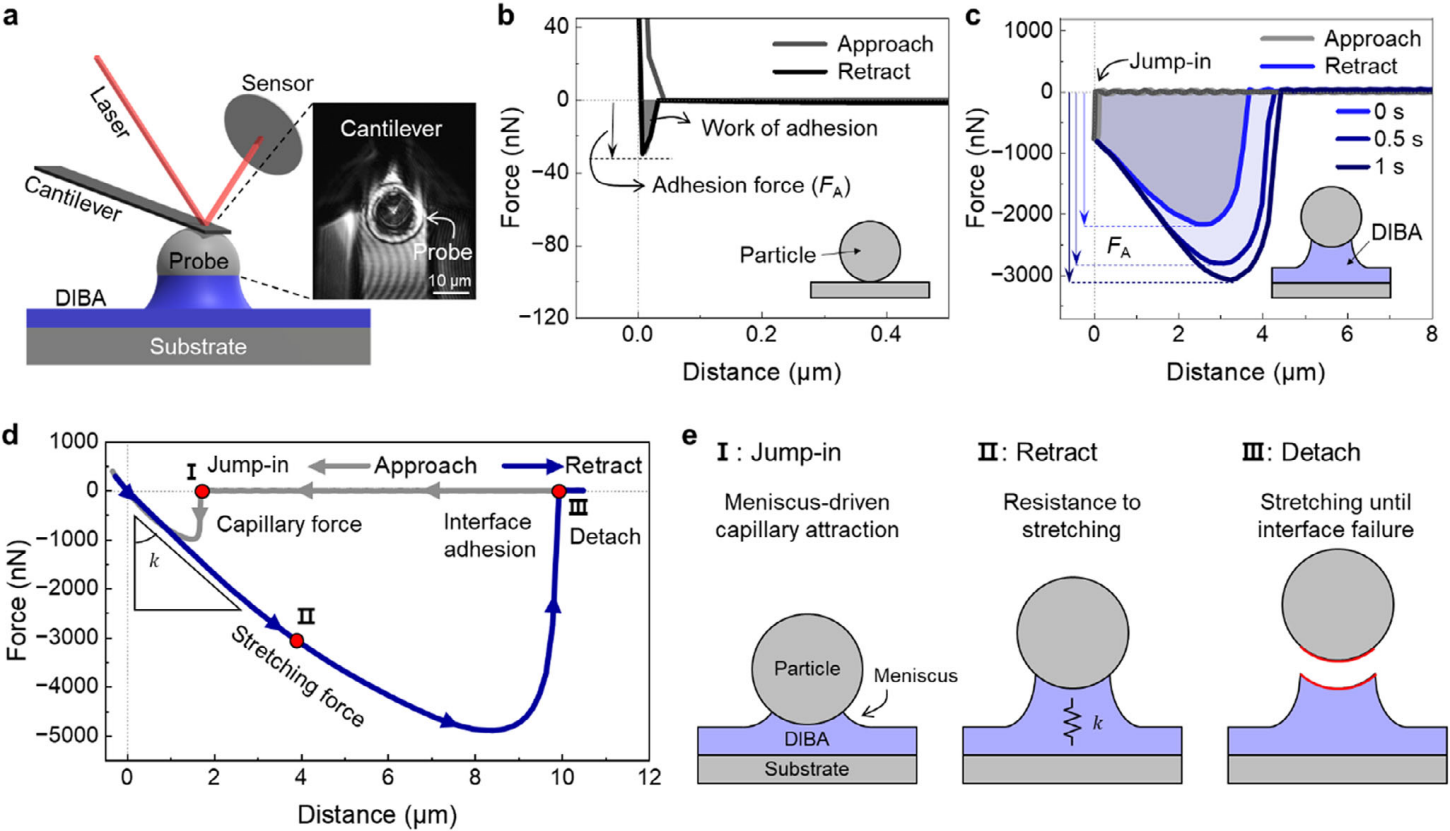
Microparticle adhesion. (a) Schematic design of the colloidal microprobe technique interacting with DIBA‐coated substrate. The inset micrograph indicates a particle adhered on edge of the tipless cantilever. (b,c) Force‐distance curves measured on (b) bare and (c) DIBA‐coated glass substrate. The arrow line indicates adhesion force, and shaded area represent the work of adhesion. (d) Force‐distance curve of DIBA‐coated glass substrate with detailed explanation. Arrows and red dots indicate the direction of probe movement and interaction events (I: jump‐in, II: retract, III: detach), respectively. (e) Schematic representation of adhesion mechanism of DIBA to microparticle.

For filter coating applications, materials that form a meniscus could exert strong capillary adhesion to particles [9, 57, 58]. To quantitatively evaluate particle adhesion, we measured the adhesion force using colloidal probe atomic force microscopy (AFM) (Figure 3a, Figure S7). This technique enables measurement of normal‐direction adhesion by approaching and retracting a particle from a substrate [59, 60]. Microparticles with a diameter of 10.0 ± 0.5 µm were selected as probes corresponding to the size of typical airborne PM (inset photo of Figure 3a). The adhesion force (*F_A_*) was considered the maximum pull‐off force required to detach the probe. As a control, we first measured particle adhesion on flat bare glass (Figure 3b), which was employed to simulate adhesion of solid filters. The adhesion force on a bare glass substrate was measured to be 31.6 ± 4.1 nN. Such solid‐solid interaction is mainly governed by van der Waals forces and occurs through point contact with the substrate. As a result, almost direct detachment from substrate was observed, and the work of adhesion required for detachment was extremely low at 0.7 ± 0.1 fJ.

We analyzed the adhesion force of DIBA using minimal contact, allowing the probe to touch the adhesive surface before immediate detachment (Figure 3c). Typically, the adhesion of a particle to a soft polymer is strongly affected by the meniscus formed via indentation [57, 61, 62]. However, since it is challenging to accurately estimate interactions of airborne particles to filters, we quantified minimum adhesion force required for detachment from DIBA. As the probe approached the surface, an attractive jump‐in contact (negative force) was observed. With immediate retraction (0 s), the adhesion force was measured to be 2,150.7 ± 31.9 nN. The DIBA displays superior adhesive capabilities, given the adhesion value is almost 70 times greater than uncoated glass substrate. In addition, the DIBA continued to pull the probe downward during detachment. Hence, the maximum force was not observed immediately during retraction. It appeared at 2.6 ± 0.2 µm from the surface and the work of adhesion is 5,828.3 ± 159.0 fJ, which is orders of magnitude higher than that of bare glass. The ability of DIBA to generate strong capillary adhesion originates from its stress relaxation behavior at room temperature (Figure 2f). Water‐mediated dissociative bond exchange allows network rearrangement that drives the polymer to wet around the particle and form a meniscus.

We also investigated how contact time affects the evolution of the meniscus and the adhesion. As illustrated in figure 3c, increasing the contact time from 0 s to 0.5 s to 1 s progressively increases both the adhesion force as well as the work of adhesion. Accordingly, the immediate retraction (0 s) represents the baseline adhesion prior to time‐dependent adhesion strengthening. This is most likely dominated by the growing meniscus and increased contact area. To qualitatively examine the time‐dependent evolution of the meniscus, a silica particle (∼300 µm), glued to a rod, was brought into contact with the DIBA surface and held in place (Figure S8). The surface energy drives DIBA to wet the particle and form a concave meniscus. The continuous rearrangement of dynamic imine bonds, as quantified through the rheological measurements, allows the DIBA to relax and flow, enabling the growth of the meniscus over time. Meniscus growth increases adhesion, which is confirmed to be significantly higher than the bare glass substrate, affording better adhesion to coated filters.

The adhesion force observed in DIBA primarily arises from three contributions: [57] capillarity from the meniscus at the contact line, deformation of the polymer network, and interfacial energy associated with the surface area. Analysis of the force‐distance curve obtained on a DIBA‐coated glass substrate under a 500 nN indentation load reveals the sequential contributions of these forces (Figure 3d). As indicated by the jump‐in event during approach (Figure 3d, step I), a meniscus forms around the particle, generating a strong capillary force. Capillarity‐driven interaction pulls the particle toward the surface and governs the initial attachment, as illustrated in the leftmost schematic of Figure 3e. Upon retraction (Figure 3d, step II), the polymer chains of DIBA resist elongation, and the probe experienced a stretching force, as depicted in the middle schematic of Figure 3e. The contribution of the stretching force is reflected in the retraction slope (*k*) of the force‐distance curve. DIBA with higher elastic modulus exhibited a steeper slope *k*, consistent with a higher stretching force (Figure S9). The final force required for complete detachment corresponds to interfacial failure (Figure 3d, step III and Figure 3g, rightmost schematic).

### 2.4 PM filtration Performances

**FIGURE 4:**
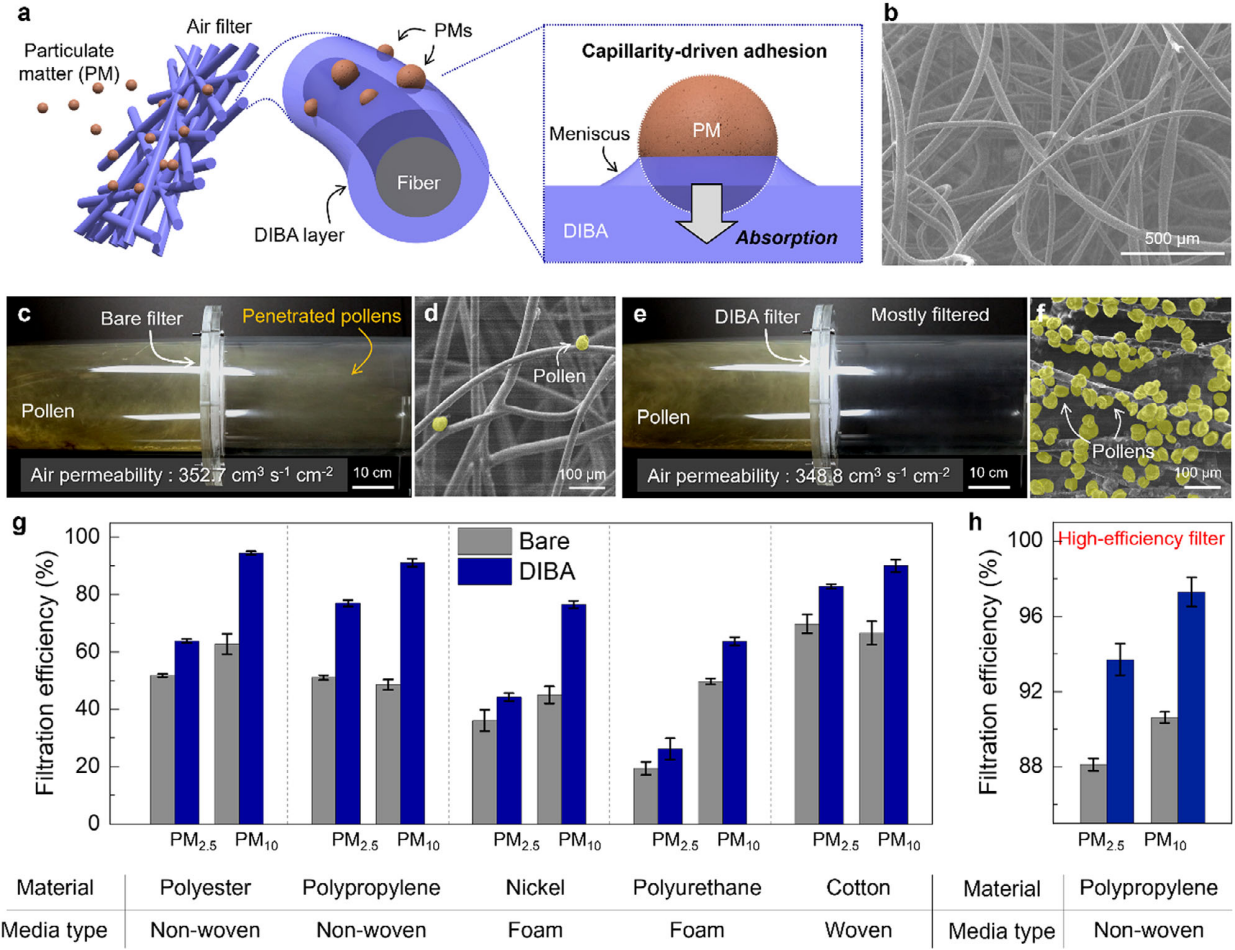
Filtration performance. (a) Schematic design of DIBA filter illustrating capillarity‐driven particle adhesion mechanism. The gray cylinder, blue layer, and brown particles represent the filter fiber, DIBA film, and particulate matter (PM), respectively. b) SEM image of polyester filter coated with a thin (< 500 nm) DIBA film at loading of ∼10 g m^−2^. (c–f) Filtering demonstration using a cylindrical dust chamber. (c,e) Snapshots of (c) bare and (e) DIBA filter immediately after exposure to pollen‐laden airflow. Air permeability was evaluated under 125 Pa following the ASTM D737 standard. (d,f) SEM images of (d) bare and (f) DIBA filter after pollen filtration. Captured pollen particles are pseudo‐colored in yellow for clarity. (g,h) Filtration efficiencies of (g) standard filters and (h) the high‐efficiency filter. Numerical details such as filtration efficiency, air permeability, and pressure drop are provided in Table S1. Sample sizes: *n* = 10 (c,e), and 3 (g,h), respectively.

We applied DIBA onto the surface of filter media (Figure 4a), which we refer to as the DIBA filter. We fabricated DIBA filters by spray coating a precursor solution onto the filter media (Figure S10). Due to the low surface tension of the dissolving solvent (i.e., toluene), the precursor solution formed a uniform layer over the filter fibers. After curing the prepolymer with crosslinker, a homogeneous, thin coating (< 500 nm) of DIBA on the filter media was formed (Figure 4b). Since the coating thickness was sufficiently thin to avoid pore blockage, the DIBA coating did not affect air permeability (352.7 ± 4.1 and 348.8 ± 4.5 cm^3^ s^−1^ cm^−2^ for bare and DIBA polyester filters, respectively). To verify the chemical stability of the crosslinked network under filtration conditions, we conducted gas chromatography‐mass spectrometry (GC‐MS) analysis to evaluate potential volatile release from DIBA, particularly low‐molecular‐weight crosslinker species. No detectable terephthalaldehyde signal was measured under the simulated operating conditions, confirming the absence of volatile emission and supporting the stability of DIBA for air filtration (Figure S11). By leveraging its superior adhesion, the DIBA filter exerts significantly improved filtering performance (Movie S1). When pollen particles (∼50 µm in diameter) were blown into uncoated polyester filters, most of the airborne particles were not captured and passed through the filter (Figure 4c). SEM analysis of the pollen‐filtered bare filter confirmed that 7.6 ± 1.4 particles mm^−2^ were captured by the filter fibers (Figure 4d). Conversely, 225.9 ± 25.0 particles mm^−2^ of the pollens were effectively captured by the DIBA filter without a change in air permeability (Figure 4e). An SEM image of the DIBA filter after filtration distinctively exhibited pollen particles adhered to fiber surfaces (Figure 4f).

In addition, we quantitatively measured the filtration efficiency by using a custom‐built dust chamber (Figure S12). This filter testing dust chamber is designed to simultaneously quantify (1) PM concentration and (2) pressure drop across the filter. Figure 4g shows the filtration efficiencies for filters of varying materials and media types with different pore sizes, and Figure 4h presents the high‐efficiency filter separately. These efficiencies are categorized into two size groups corresponding to inhalable PM: PM_2.5_ (0.3–2.5 µm) and PM_10_ (2.5–10 µm). Gray bars in Figure 4g,h represent the efficiencies of bare filters, while blue bars represent DIBA filters. The DIBA formed a stable adhesive layer on a wide range of filter and filter‐applicable medias, such as non‐woven polyester, polypropylene media, nickel foam, polyurethane foam, and woven cotton. The photographs and SEM images of each filter medium are presented in Figure S13 (Supporting information). Across all these materials, we observed notable improvements in efficiencies of 10–30% by coating the thin DIBA layer compared with the bare counterparts. Notably, the viscoelastic DIBA filters demonstrated performance improvement comparable to liquid‐based PRO filters (Figure S22) [11]. More importantly, because efficiency improvement was achieved without any increase in pressure drop, the overall quality factor (QF) was also increased (Figure S14). Besides, DIBA forms a mechanically stable solid film that enables precise control over film thickness. As the coating amount increases, partial pore clogging leads to a gradual rise in pressure drop; however, at a loading level of ∼10 g m^−2^, the pressure drop remains within approximately 10% of the bare filter while maintaining high filtration efficiency (Figure S15). To evaluate the stability of the water‐mediated dynamic network under external conditions, DIBA filters were stored under different humidity (10%, 50%, and 90% RH) and temperature conditions prior to testing (Figure S16). No significant change in filtration performance was observed after humidity storage, whereas thermal drying increased modulus and reduced filtration efficiency; nevertheless, enhanced performance relative to bare filters was retained below 200°C, defining the practical operating range of DIBA. The superior filtration performance of DIBA filters was further validated by the Friend of Industry Technology Information (FITI) Testing & Research Institute, an internationally accredited testing organization under the Korea Laboratory Accreditation Scheme (KOLAS) (Figure S17). For the minimum efficiency report value (MERV) that indicating industry standard for air filter, the bare filter exhibited a MERV 6 rating, corresponding to a conventional pre‐filter level. After DIBA coating, the same filter media was upgraded to MERV 11 rating that reaching a medium‐grade filter. This substantial enhancement demonstrates that the DIBA layer can improve the filtering performance of commercially available filtration media.

### 2.5 Ultrafast PM Filtration

**FIGURE 5:**
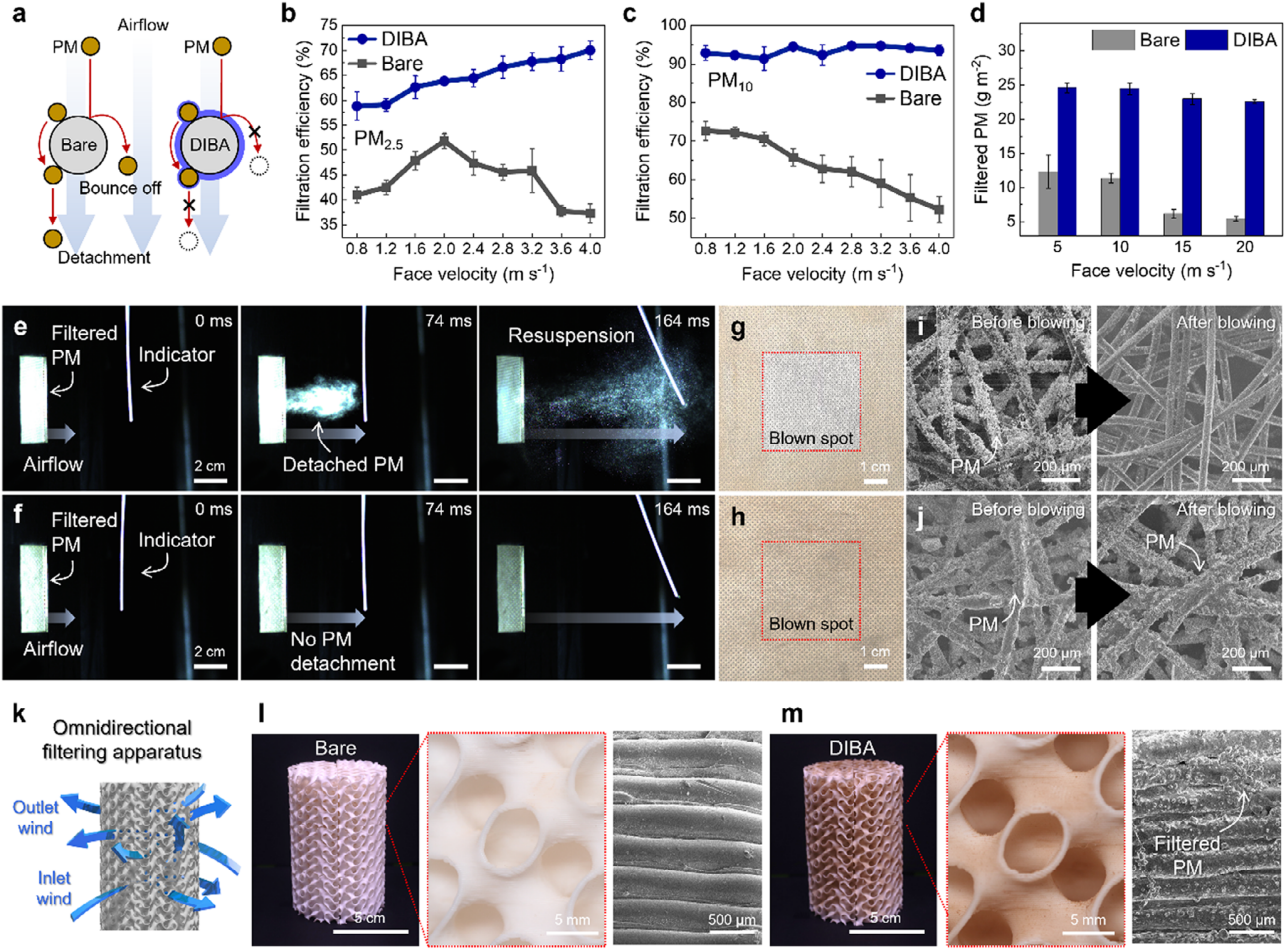
Capillarity‐mediated filtering characteristics: ultrafast and omnidirectional filtration. (a) Schematic illustrating PM filtering of bare (left) and DIBA (right) fibers under fast airflow. (b,c) Filtration efficiencies of bare (gray) and DIBA (blue) polyester filters for (b) PM_2.5_, and (c) PM_10_ as a function of face velocity using particle counter. (d) Filtering performance of filters under ultrafast (over 5 m s^−1^) conditions by comparing quantity of filtered PM over filter media. (e,f) High‐speed camera images of PM‐filtered (e) bare and (f) DIBA polypropylene filters when airflow in the reverse direction is applied. (g,h) Photographic images of (g) bare and (h) DIBA filters after a reverse airflow test. The red dotted square indicates the spot directly impacted by the reverse airflow. (i,j) SEM images of (i) bare and (j) DIBA filters before and after resuspension. Captured PMs on the filter fiber are indicated with white arrows. (k) Schematic of the 3D‐printed gyroid filtering apparatus used for omnidirectional filtration. (l,m) Photographic (left, middle) and SEM (right) images of (l) bare and (m) DIBA filtering apparatus after omnidirectional filtration using multiple fans at PM‐dispersed chamber. *n* = 3 for (b), (c), and (d).

DIBA can operate reliably under ultrafast airflow conditions. In fact, as the face velocity of airflow increases, the probability of PMs interacting with filter fibers rises, thereby increasing filtration efficiency [1]. However, once the drag force of airflow surpasses the adhesion, captured PMs can detach from fiber surface (Figure 5a). Such adhesion failure leads to less efficient filtering at faster airflow. We confirmed that the efficiency of a conventional polyester filter generally diminishes beyond a certain airflow speed (gray line in Figure 5b,c). In contrast, the efficiency of DIBA filters increased at higher airflow velocity for both the PM_2.5_ and PM_10_ (blue line in Figure 5b,c), while the pressure drop remained comparable to the bare filter across all face velocities (Figure S18). Notably, even at a high velocity of 4 m s^−1^, the efficiency of DIBA for PM_10_ was approximately 40% higher than the bare filter. Moreover, we then elevated the airflow velocity even more to investigate filtering capacity under extreme conditions (above 5 m s^−1^). Because the particle counter does not provide stable PM readings above 5 m s^−1^, the relative performance was determined by quantifying the captured particles on each filter media after filtration. Due to detachment of captured PM at faster airflow, the amount of PM retained on the bare filter gradually decreased from 12.3 ± 2.4 to 5.5 ± 0.3 g m^−2^ (gray bar in Figure 5d). The DIBA filter demonstrated a stable filtering of approximately 20 g m^−2^. Captured PM on DIBA filter exhibited a relatively small change even as the airflow velocity was increased to 24 m s^−1^. A small, systematic decrease was observed as velocity increased, suggesting the possibility of some elastic bouncing of particles [1, 63, 64]. Nevertheless, the overall results demonstrate the robustness and filtering capabilities of DIBA under ultrafast airflow.

### 2.6 Suppressed PM Resuspension and Omnidirectional Filtration Characteristic

Conventional filtration systems often fail to securely retain captured PMs, leading to a resuspension into ambient air. For example, by blowing air through a PM‐filtered conventional filter with an air gun, the captured PMs were detached from the filter media, observed by a high‐speed camera (Figure 5e, Movie S2). The resuspended PM scattered the light, which made them visible as white spots. We confirmed the direction of airflow using the deflection of a wind indicator. For the DIBA filter, captured PMs remained within the polymer matrix and virtually no PM detachment was observed under the same conditions (Figure 5f). Photographic images of the conventional filter demonstrated a color change after blowing, as the brown area turned white once particles were detached (Figure 5g). In contrast, the DIBA filter retained its uniform brown color even after the same air blowing (Figure 5h). The SEM images further corroborated this finding (Figure 5i,j); the PM detached from the conventional filter fibers, whereas the PM remained securely adhered to the DIBA filter.

Conventional solid filters are limited to unidirectional operation, since captured PMs can be easily resuspended by external airflow. However, the superior adhesion of DIBA enables the filter to securely capture PMs even under omnidirectional airflow. We selected a gyroid structure as the filtering apparatus due to its key properties: a high surface area and an open network that accommodates airflow from all directions (Figure 5k). We 3D‐printed a gyroid structure with a unit cell size of 8 mm using a layer height of 0.2 mm and subsequently applied an approximately 500 nm DIBA layer by spray coating. Under the random airflow of multiple fans containing A2 standard dust (see Figure S19), the bare filtering apparatus captured almost no PMs (Figure 5l). The structure turned slightly brownish, but SEM analysis found almost no PMs on the surface since captured PMs were quickly detached by the irregular airflow. In contrast, the DIBA filtering apparatus effectively captured PMs from all directions and retained them even under subsequent turbulence (Figure 5m). The surface became visibly contaminated, and SEM images clearly confirmed high‐density particle absorption. These results suggest that DIBA can serve as a next‐generation, passive filtering solution for complex environments without requiring an external power source.

### 2.7 Particle Absorption Behavior

**FIGURE 6:**
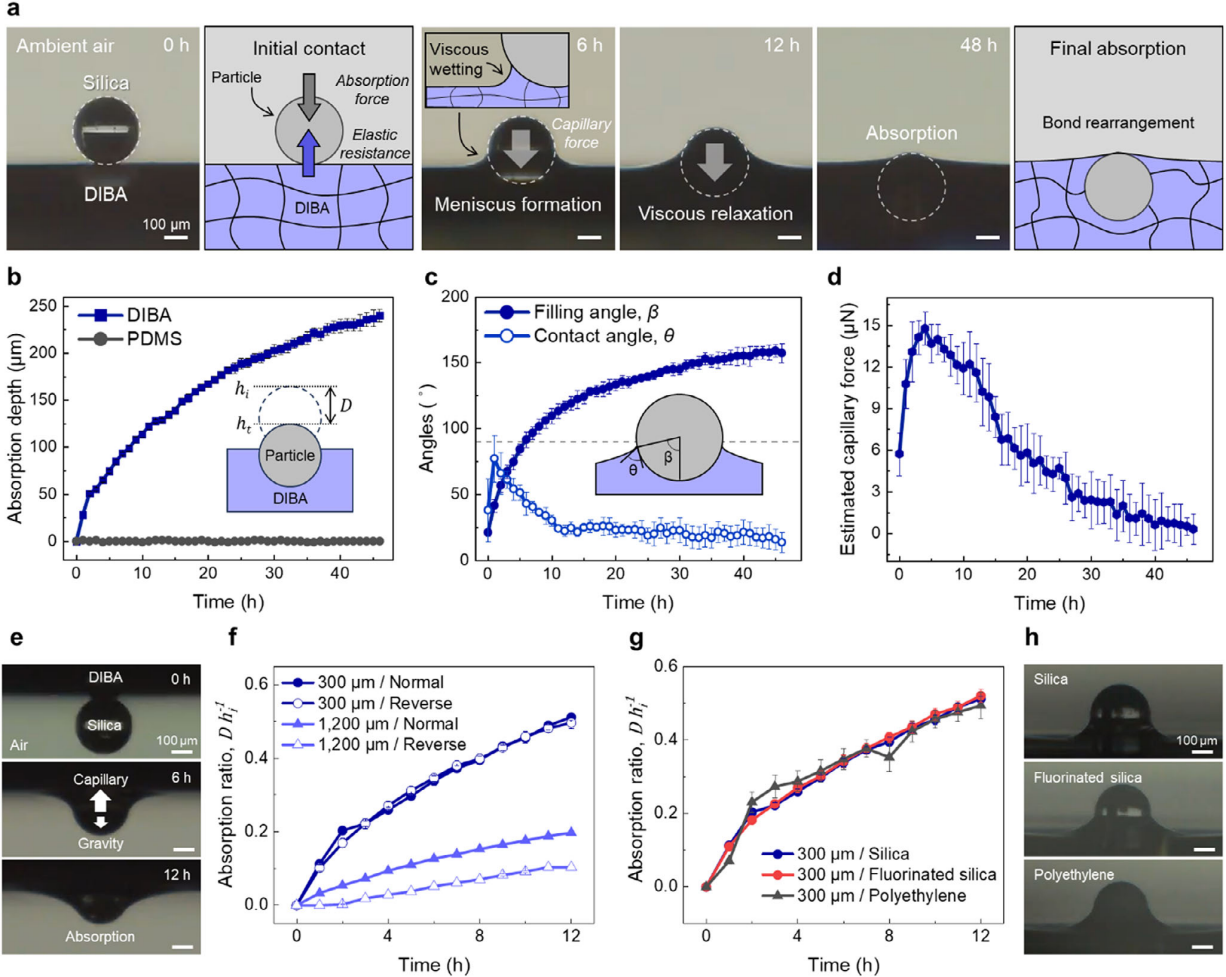
Microparticle absorption. (a) Snapshots of silica microparticle (∼300 µm) absorption behavior of DIBA. Schematics on the right of images of 0 and 48 h illustrate the interacting behavior at each state. (b) Absorption depth *D* of particle in contact with DIBA (blue) and PDMS (gray) as a function of time. Snapshots for particle absorption of PDMS are depicted in Figure S20. (c) Filling angle β (closed circles) and contact angle θ (open circles) of meniscus during particle absorption. (d) Estimated capillary force exerted on the particle during absorption. (e) Images during silica microparticle (∼300 µm) absorption of DIBA in reverse direction (gravity‐resisted). (f) Absorption ratio ($D\;h_{i}^{-1}$) of ∼300 µm (blue) and ∼1,200 µm (purple) silica particles in gravity‐assisted normal (closed symbol) and gravity‐resisted reverse (open symbol) direction. (g) Absorption ratio of bare silica (blue), fluorinated silica (red), and polyethylene (gray) particles sized of ∼300 µm. (h) Images of the absorption of DIBA for various particles from (g) at 12 h. *n* = 5 for (b), (c), (d), (f), and (g).

DIBA possesses a unique adhesion mechanism that actively absorbs particles into its polymer matrix (Figure 6a, Movie S3). When a particle first contacts the DIBA, it remains on the surface due to the elastic‐like resistance of the polymer network, while simultaneously experiencing an inward stress (e.g., capillary force, gravity). Thermodynamically, DIBA is a silicone‐based material that should have a contact angle of 0° with glass. Hence, a meniscus forms as the DIBA attempts to increase the contact area, which is being resisted by the viscoelastic properties. In the limit of zero network elasticity, the particle would be fully cloaked or fully absorbed by the polymer. The concave curvature of the meniscus generates a net inward capillary force that pulls the particle into the matrix. The local stress induced by the absorbed particle is then gradually relaxed through the dynamic bond exchange, similar to the bulk stress relaxation experiment in Figure 2f. As the stress continues to relax, this leads to gradual absorption of the particle. After the particle is fully absorbed, the surrounding polymer chains rearrange to reach an equilibrium configuration. The particle is ultimately absorbed within the dynamic network. To elucidate the underlying mechanism of particle absorption, we analyzed the absorption depth (*D*) and the meniscus profile as a function of time. The absorption depth *D* is defined as the difference between the initial contact height (*h_i_*, i.e., particle diameter) and the instantaneous particle height after absorption (*h_t_*). The permanently crosslinked PDMS with comparable storage modulus (G′) to DIBA, which behaves as a purely elastic solid, only permitted surface adhesion of the particles, as depicted by the gray line in Figure 6b (Figure S20). The DIBA demonstrated active particle absorption, and its penetration rate was characterized by a rapid initial phase followed by a gradual slowdown (blue line in Figure 6b). Absorption trend of DIBA can be interpreted using meniscus geometry and its corresponding absorption force. The driving mechanism for absorption is the thermodynamic wetting, which in equilibrium has a 0° contact angle (i.e., full spreading). This wetting induces formation of a meniscus, which in term generates a capillarity force associated with particle absorption. To verify this, we estimated the magnitude of the capillary force as a function of time by first analyzing optical images of the meniscus (Figure 6c). The capillary force on a sphere is given by *F_C_* =  πγ*d*sin (β)sin (β + θ), where γ is the surface tension, *d* is the particle diameter, θ is the meniscus contact angle and β is the filling angle that defines the position of the three‐phase contact line on the particle [9, 65]. As particle absorption progresses, the filling angle β gradually increases, exhibiting a similar trend with the absorption kinetics. In contrast, the contact angle θ gradually decreases toward 0° as the meniscus becomes flattened. Figure 6d represents an estimated capillary force calculated using the measured angles, assuming the surface tension of the network is comparable to the prepolymer state (γ_*DIBA*_ ≈ γ_*AmPDMS*_). The rapid initial absorption is attributed to the strong capillary force with an estimated peak value of approximately 15 µN. As the particle is absorbed through DIBA relaxation, the meniscus flattens. This in turn weakens the vertical component of the capillary force acting on particle. Additionally, as the particle becomes further embedded, the volume of the polymer network undergoing stress relaxation also expands. The deceleration is likely due to two combined effects: (1) the stress relaxation needs to occur over a larger network volume, which is a slower and (2) the driving force decreases as the particle becomes engulf, since there is more wet area and the contact angle approaches 0°. Therefore, the penetration rate reflects the competition between two main factors: The driving force due to wetting, which induces a capillary force, and the viscoelastic resistance and relaxation of the rearranging network.

Gravity also contributes to the downward force for particle absorption. However, for a ∼300 µm silica particle, the gravitational force (∼0.2 µN) is negligible since it is approximately two orders of magnitude smaller than the capillary force. To investigate the influence of gravity on the absorption, we inverted the DIBA substrate and observed the particle being absorbed upwards against the gravity (Figure 6e). We found that the absorption rate of the reverse (gravity‐resisted) direction remained nearly identical to the normal (gravity‐assisted) direction for up to 12 h (Figure 6f). This result indicates that the gravity effect on absorption was negligible at the micron scale. To further confirm the scale‐dependent effect, we repeated the experiment with a much larger ∼1,200 µm diameter silica bead. To facilitate an intuitive comparison despite the difference in particle size, we converted the absolute absorption height into an absorption ratio ($D\;h_{i}^{-1}$). In this case, the influence of gravity was much larger, and a clear difference in absorption ratio was observed between the normal and reverse directions. It implies that absorption into the polymer can occur regardless of the capture direction when dealing with micron‐scale particles. Furthermore, DIBA can absorb any particle regardless of surface energy. Absorption using hydrophobic particles such as fluorinated silica and polyethylene (PE) revealed that their absorption rates were nearly identical to hydrophilic silica particles (Figure 6g). Negligible change in the absorption trend can be attributed to the inherently low surface energy of PDMS chains in DIBA. Due to its hydrophobic nature, DIBA can effectively wet even hydrophobic surfaces to form a stable meniscus (Figure 6h). Similar dynamic imine‐based polymers have been reported to spread even on hydrophobic substrates [66]. Therefore, DIBA maintains its absorption mechanism regardless of particle surface energy.

Crosslinking density is a key parameter determining the particle absorption behavior. A higher crosslinking density increases the elastic modulus, which reduces polymer chain mobility and requires a longer time for stress relaxation. As a result, the viscoelastic flow required for particle absorption is suppressed, and the absorption rate decreases significantly (Figure S21). Consistently, absorption was delayed both when residual water was removed by drying and when the crosslinker ratio was increased. Furthermore, this kinetic difference also affects the filtering performance (Figure S4). DIBA with significantly higher crosslinking density displays more elastic‐like properties. This causes captured particles to cover the adhesive layer, making further filtering dependent on particle‐particle interactions rather than particle‐DIBA interactions. Hence, it can also increase the probability of an elastic bounce before particles can be absorbed [1, 63, 64].

### 2.8 Particle Absorption Capacity

**FIGURE 7:**
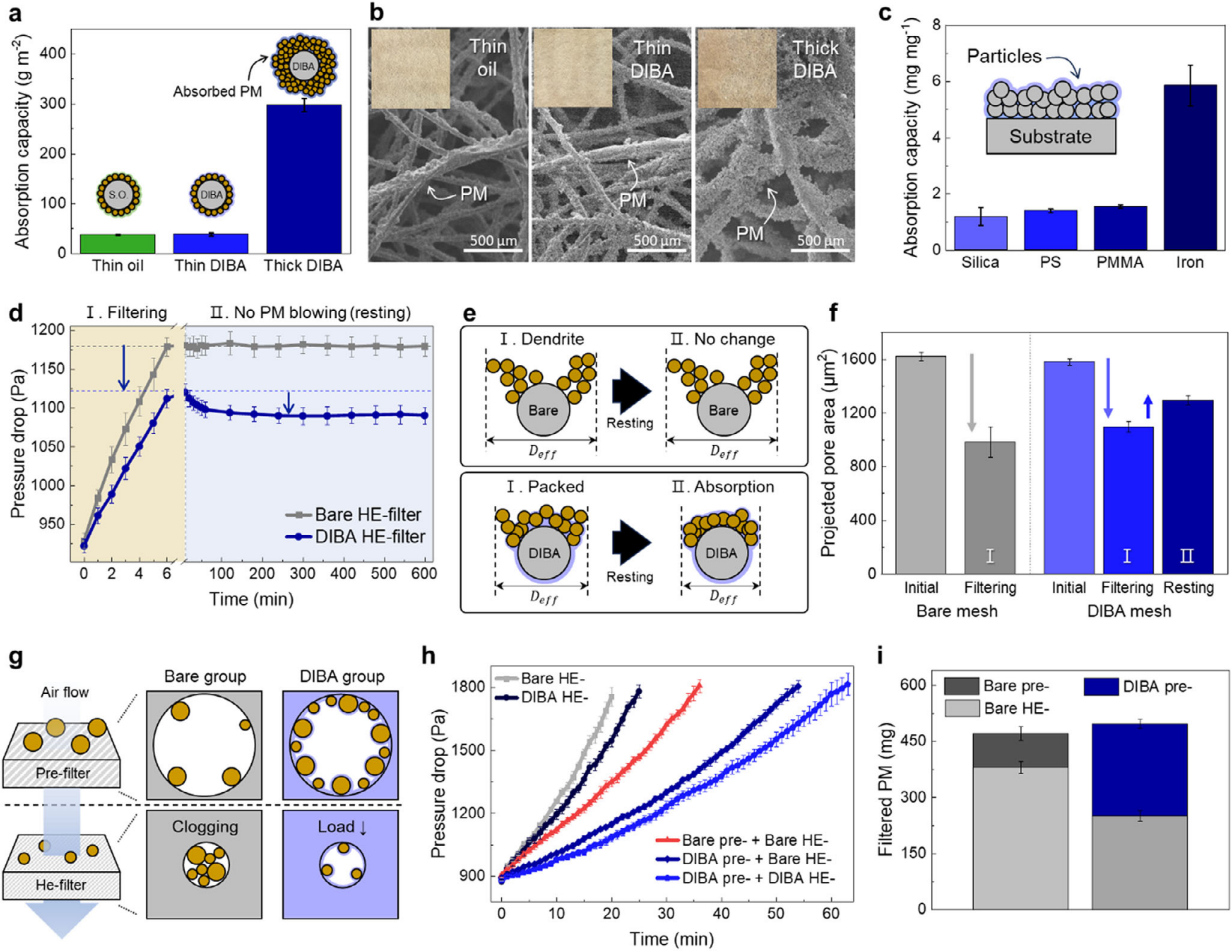
Absorption capacity and pressure drop. (a) Absorption capacity of thin oil (green), thin DIBA (blue), and thick DIBA (dark blue) coated polyester filters for A2 dust. (b) SEM and inset photographic images of thin oil (left), thin DIBA (middle), and thick DIBA (right) filter after maximum absorption of A2 dust. (c) Absorption capacity of DIBA for various particle types. Silica (light blue), PS (blue), PMMA (dark blue), iron powder (dark navy) is indicated. (d) Pressure drop curves of bare (gray) and DIBA (blue) HEPA filters (1) during filtration and (2) during the subsequent relaxation phase (no PM blowing). Blue arrows indicate pressure drop difference at each filtering step. (e) Schematic structure of captured PMs on bare (top) and DIBA (bottom) fibers. The term *D_eff_* represents the effective diameter of PM captured filter fibers. (f) Projected pore areas of bare (gray) and DIBA (blue) metal mesh after filtration and then resting of 24 h for the case of DIBA metal mesh. All samples were loaded with approximately 80 g m^−2^ of PM. (g) Schematic design multi‐stage filtration of bare (left) and DIBA (right) pre‐ and HE‐filter group. (h) Pressure drop curves as a function of PM filtration time. (i) Weights of filtered PMs on bare (dark gray) and DIBA (blue) pre‐filters combined with bare HE‐filters (light gray box). *n* = 3 (a), (c), (d), (h), and (i), 6 (f).

Conventionally, filters can re‐contaminate the purified air because captured PMs do not securely adhered on fiber. In contrast, adhesion‐enhanced filters immobilize captured PM, which increases the capacity to retain particles without causing re‐contamination. We deposited A2 standard dust onto the adhesive‐coated filters, then removed unretained particles using an air gun after 24 h of absorption. The absorption capacity was defined as the mass at which no further dust absorption occurred, and the mass of captured PM reached a plateau. We confirmed that a filter coated with a ∼509 nm‐thick DIBA thin film (loading: 13.4 ± 1.2 g m^−2^) captured 38.7 ± 3.5 g m^−2^ of PM (light blue bar in Figure 7a). Absorption capacity of a DIBA thin film is comparable to a previously developed PRO filter coated with a ∼539 nm‐thick silicone oil thin film (loading: 14.2 ± 1.6 g m^−2^), which retained 38.9 ± 2.2 g m^−2^ (gray bar in Figure 7a). The viscoelastic flow behavior allows DIBA filters to absorb particles through a mechanism analogous to a liquid‐coated filter. A limitation of true liquid oil‐coatings is its instability at increased film thickness due to the Plateau‐Rayleigh instability [12, 13, 14]. This instability causes the liquid film to break up into droplets that leads to a higher pressure drop and liquid detachment under airflow (Figure S22). In contrast, since a crosslinked network provides structural integrity, DIBA can be coated as a stable bulk film on filter fibers. Even with a ∼3.5 µm‐thick film (loading: 97.0 ± 2.1 g m^−2^), we confirmed a negligible change in pressure drop and no delamination of the coated layer. The thick DIBA film absorbed 294.7 ± 15.8 g m^−2^ of PM, which is approximately 8 times higher than the thin film‐coated filters (blue bar in Figure 7a). SEM images and inset photograph visually corroborate the quantitative analysis of absorption capacity (Figure 7b). The bulk DIBA film accumulated substantially more PM and developed a much thicker fiber, whereas thin DIBA and thin oil coating showed comparable PM loading, consistent with their similar absorption capacity. Additionally, DIBA can capture a broad range of particles (Figure 7c). To quantify the maximum absorption capacity of DIBA, we prepared a DIBA film‐coated glass substrate with a film thickness of 14.0 ± 2.0 µm and measured its particle absorption and quantified the maximum quantity of particles that can be absorbed per unit mass. We confirmed that DIBA absorbed a wide variety of particles, including silica, polystyrene (PS), polymethyl methacrylate (PMMA), and iron powders. Cross‐sectional SEM observations revealed that the particles were continuously absorbed and formed multilayered arrangements within the matrix (Figure S23).

### 2.9 Delay Effect of Pressure Drop Increase by Filtration

The accumulation of PM leads to clogged pores and increases the pressure drop across filter media [1]. The lifetime of an air filter is defined as the time required for the pressure drop to reach twice its initial value, which is considered the critical threshold for maintaining operational efficiency. Here, the absorption behavior provides advantages in extending the lifetime of the DIBA filter. We measured the pressure drop trend during filtration for 6 min and rested without blowing additional PM to confirm the absorption effect on pressure drop. Filters with smaller pore sizes (e.g., high‐efficiency (HE) filter) experience a pressure drop increase more significantly. In conventional filters, PMs accumulate on other PMs forming a dendritic structure (Figure 7e top) that rapidly increases the pressure drop during filtration (gray line in Figure 7d). Under the identical filtration conditions, the DIBA HE‐filter demonstrates a 5.7% lower pressure drop compared with a bare HE‐filter (blue line in Figure 7d). After the PM blowing was stopped, the pressure drop of the DIBA filter decreased even further, whereas that of the bare filter remained unchanged. Decrease in pressure for the DIBA filter occurs because the captured dust was gradually absorbed into the polymer matrix, thereby reopening the pores (Figure 7e). In conventional filters, PMs aggregate to form a dendritic structure with a large effective diameter (*D_eff_*) that is maintained after filtration. Planar metal mesh demonstrates a decrease in pore size because of captured PMs (Figure 7f, Figure S24). It exhibited a reduction in the projected pore area from 1,621 ± 31 to 982 ± 112 µm^2^ (gray bars in Figure 7f). In contrast, PMs captured on the DIBA filter are densely packed through capillary‐driven packing and subsequent absorption into the matrix (Movie S4). High‐density packing results in a smaller effective diameter *D_eff_* for the accumulated PM. Even with the same PM loading (∼80 g m^−2^), the projected pore area is reduced only to 1,097 ± 39 µm^2^ and subsequently recovered 1,295 ± 35 µm^2^ after 24 h of absorption, which is approximately 34% more open than the bare mesh (blue bars in Figure 7f).

Because HE‐filters have small pore sizes, they are susceptible to clogging by large PMs and consequently lead to rapid increases in pressure drop. A pre‐filter that screens large PMs using its relatively thick fibers and larger pore structure is usually placed upstream to reduce PM loading on the HE‐filter (Figure 7g). A bare HE‐filter placed downstream of a bare pre‐filter (red line in Figure 7h) exhibited an approximately 80% slower rate of pressure drop increase compared to its stand‐alone HE‐filter (gray line in Figure 7h). Here, an efficient DIBA pre‐filter was even more effective at suppressing the pressure increase compared with the bare pre‐filter (dark blue line in Figure 7h). To better understand the screening effect of the pre‐filter, we measured the mass of filtered PMs on each filter group (Figure 7j). Because the HE‐filter operates at approximately 99% filtration efficiency, the total mass of PMs filtered over the identical PM‐loading period was nearly identical for both filter groups. However, the distribution of these filtered PMs between the pre‐filter and the HEPA filter differed. The DIBA pre‐filter captured 169% more PMs than its bare counterpart and reduced the PM load on the downstream HE‐filter by 34%. As a result, DIBA filter group shows the longest operational lifetime. Indeed, the time required to double the pressure was 75% longer for the DIBA group than for the bare group, suggesting that DIBA filters offer approximately 2 times longer lifetimes.

## 3 Conclusion

We addressed the weak particle adhesion of conventional filters with dynamic imine bond adhesive (DIBA) coatings, comprised of a polymer network crosslinked with dynamic imine covalent bonds. Viscoelasticity of the DIBA layer provides both mechanical stability and capillarity‐mediated strong particle adhesion. The viscoelastic, DIBA‐coating allows filters to effectively capture PM even in ultrafast airflow environments up to 20 m s^−1^, opening new possibilities for high‐speed filtration. Since DIBA allows for the absorption of surface‐adhered PMs, filtered particles are strongly anchored inside polymer matrix. This anchoring of captured PMs prevents resuspension, enabling effective omnidirectional filtration for outdoor environments with high PM levels with natural winds. Counterintuitively, although DIBA captures more PMs, the dense packing of the absorbed particulates slows the increase in pressure drop and extends the lifetime of the filter compared to conventional, uncoated counterparts. This dynamic covalent polymer‐based filter platform, which combines capillary adhesion with continuous particle absorption, presents a promising new strategy for developing next‐generation adhesive filters with both high mechanical stability and extended operational lifetime.

## 4 Experimental Section

### 4.1 Fabrication and Characterization of Dynamic Imine Bond Adhesive

The precursor solution for synthesizing the dynamic imine bond adhesive (DIBA) was prepared by combining an amine‐modified polydimethylsiloxane prepolymer, aminopropylmethylsiloxane‐dimethylsiloxane copolymer (AmPDMS, Gelest), with terephthalaldehyde (99%, Sigma‐Aldrich). AmPDMS contains 6–7 mol% aminopropylmethylsiloxane units, corresponding to approximately 65 total siloxane repeat units per chain, of which about 4 amine functionalities. Terephthalaldehyde serves as a difunctional aldehyde linker that forms imine bonds with the primary amines on AmPDMS. Because terephthalaldehyde is provided as a solid powder, it was first dissolved in toluene (99%, Samchun Chemicals) at 25 mg mL^−1^. Crosslinker solution was subsequently mixed with the AmPDMS prepolymer to form the precursor mixture used for imine network formation. The mixture was left at ambient conditions for 24 h to allow the imine coupling reaction to proceed. During this time, the solvent evaporated gradually under ambient conditions. The partially cured mixture was further dried in a vacuum oven to remove residual solvent and control the water content in the polymer matrix.

The precursor solution was diluted with toluene to 10 wt.% and then spray‐coated onto the filters using a 0.35 mm nozzle at a spraying pressure of 0.5 MPa. At a reduced concentration of 10 wt.%, the solution remained uncrosslinked to ensure spray because high solvent fraction prevented network formation. Under this condition, crosslinking occurred after deposition when solvent evaporation on the substrate or filter surface enabled the formation of the DIBA network. Crosslinking of DIBA on both substrates and filters were analyzed by Fourier‐transform infrared (FTIR) spectroscopy (FTIR‐4600, Jasco) within the range of 4000–600 cm^−1^, using 32 scans at a resolution of 4 cm^−1^. Incorporation of DIBA within the filter fibers was demonstrated through energy‐dispersive X‐ray spectroscopy (EDS) performed on a field‐emission scanning electron microscope (FE‐SEM, SIGMA, Carl Zeiss), as depicted in Figure S10. The thickness of the DIBA layer was estimated by calculating the total surface area of the filter fibers based on average fiber diameter and the total polymer volume derived from the filter mass and density. The fibers were approximated as uniform cylinders to estimate the total fiber length. The applied DIBA volume was determined from its mass and density. Assuming the oil formed a conformal hollow cylindrical coating around each fiber, the coating thickness was calculated from the corresponding volume balance. Gas chromatography‐mass spectrometry (GC‐MS, TSQ 8000 Evo, Thermo Fisher Scientific) analysis was performed to determine whether any volatile organic components were present in DIBA after incubating the sample at 30°C for 1 h. Differential scanning calorimetry (DSC) measurements were performed at heating rate of 5°C min^−1^ over a temperature range of ‐90°C to 150°C under a nitrogen atmosphere. Thermogravimetric analysis (TGA) was conducted at heating rate of 5°C min^−1^ from 25°C to 800°C under nitrogen flow.

All rheological measurements were performed using a rheometer (HR20, Trios). Owing to the low stiffness of the samples, an axial force of 1 N was applied during all shear measurements. All rheological measurement was operated under 25°C. A parallel‐plate geometry with an 8 mm diameter was used for standard shear tests. Amplitude sweeps were performed with a strain ranging from 0.1 to 100% at an angular frequency of 1 Hz. Frequency sweep tests were conducted within the linear viscoelastic region (LVR) by applying a strain of 1% over an angular frequency range of 0.1–100 rad s^−1^. Creep‐recovery measurements were conducted under a shear stress of 100 Pa with creep for 600 s and recovery for 1800 s. Stress relaxation behavior was evaluated by imposing an instantaneous 1% strain and recording the relaxation behavior for 6 h.

### 4.2 Microparticle Adhesion Characterization

Adhesion forces were measured using a colloidal microprobe atomic force microscopy (AFM, NanoWizard, JPK Instruments). A silica microparticle (10.0 ± 0.5 µm, Cospheric) was fixed onto a tipless cantilever (MikroMasch) using epoxy glue. Prior to particle attachment, spring constant and deflection sensitivity of the cantilever were calibrated using non‐contact method. Force‐distance spectroscopy was performed with approach and retraction speeds of 2 µm s^−1^. To ensure mechanical contact with the substrate, indentation up to 500 nN was applied before retraction. For DIBA samples, adhesion was characterized under two contact conditions: indentation contact and surface contact. The surface contact condition with minimal indentation was achieved by approaching the substrate in 10 nm increments using the piezo stepper. All adhesion measurements using colloidal microprobe AFM were conducted on planar samples prepared by spin coating the precursor solutions onto cover glass substrates at 2,000 rpm for 60 s to ensure consistency. Force‐distance curves were interpreted by monitoring the deflection of the cantilever. As detailed in Figure S7, the analysis of the force curve proceeded through the following stages: (I) approach toward the DIBA‐coated substrate, (II) onset of contact with DIBA, (III) meniscus formation and capillary attraction, (IV) retraction with meniscus stretching, and (V) meniscus rupture and probe detachment. The maximum force observed during the retraction was interpreted as the adhesion force required for detachment, and the integrated area of the retraction curve was calculated as the work of adhesion.

Meniscus formation of DIBA on a microparticle was visualized using a MicroHD camera (Eakins). The DIBA film was prepared by drop‐casting the precursor solution for 200 µL onto cover glass (Marienfeld) substrate. A silica particle (296.8 ± 21.3 µm) was bonded to the tip of a 300 µm diameter stainless steel rod using epoxy glue and cured for 24 h to ensure firm attachment. The particle‐attached rod was then connected to a load cell (LSB200, Futek) and mounted onto a motorized z‐stage (SMZ‐80100‐x, Science town). The z‐stage was used to bring the particle into contact with the substrate, and the onset of particle‐surface contact was determined from the first detectable change in the load cell signal. Stage motion was stopped immediately upon surface contact, and the meniscus formation was imaged. Meniscus profiles were subsequently extracted from the images by outlining the interface using ImageJ.

### 4.3 Filtering Performance

Visual demonstration of filtering was conducted using a cylindrical wind chamber with a diameter of 15 cm. An airflow was generated inside the chamber using a fan, and commercially available pine pollen particles were introduced into the stream. The trajectory of pollen particles were imaged using a camera (EOS 80D, Canon). The pollen adhered to the fiber surface was demonstrated using a tabletop scanning electron microscope (SNE‐4500 M Plus, SEC). The pollen particles in the SEM micrographs were digitally pseudo‐colored for clarity.

Quantitative filtration performance was measured using a custom‐built air filter testing chamber that complies with the DIN 71460–1:2006 standard. The reliability of this custom setup had been verified in our previous study conducted in collaboration with the certified testing agency (KITECH) [11]. In addition, the performance of the DIBA filter was independently assessed by Friend of Industry Technology Information (FITI) Testing & Research Institute in accordance with the ANSI/ASHRAE 52.2 standard, including measurement of minimum efficiency reporting value (MERV) and overall filtration efficiency. Aerosol concentrations were measured using a particle counter (11‐A, GRIMM) capable of detecting particles in the 0.25‐32 µm range. The particulate matter (PM) was A2 standard dust (Powder Technology) with a size distribution spanning 0.3‐10 µm. Aerosol was dispersed into testing chamber by feeding N_2_ gas through the particle generator (SAG 410, TOPAS). Filtration efficiency (FE) was calculated using *FE*  =  (*C_b_* − *C_a_*) *C_b_* ^−1^, where *C_b_* and *C_a_* represent PM concentrations before and after filtration, respectively. FEs were categorized into PM_2.5_ (0.3–2.5 µm) and PM_10_ (2.5–10 µm). The pressure drop (ΔP) across the filter media was recorded using a pressure gauge (PTA202D, SCS). As ΔP increased, the system automatically adjusted the face velocity using airflow data obtained from an orifice meter (CP213, KIMO). The effective filtration area was set to 100 cm^2^. All filter media were neutralized to remove electrostatic charge using ion blower (DS‐150B, DOOSTEC) to avoid unintended charge‐induced interactions. A quality factor (QF) was calculated from FE and ΔP to compare overall filtration performance; it is defined as *QF*  =   − ln (1 − *FE*) Δ*P* ^−1^. Tested filter media included commercially available non‐woven polyester, polypropylene, nickel foam, urethane form, woven cotton, and high‐efficiency non‐woven polypropylene filters. Pore structures were characterized by SEM, and air permeability was measured using an air permeability meter (ADE‐580A, AND) following ASTM D737 (125 Pa, 38 cm^2^).

### 4.4 PM Resuspension and Omnidirectional Filtration

Resuspension of captured PM was recorded using a high‐speed camera (FASTCAM Mini AX100, Photron) operating at 500 frames per second. The PM‐captured non‐woven polypropylene filter was mounted onto a zig holder providing an open area of 25 cm^2^. N_2_ gas was blown from the opposite side of the dust‐loaded surface using air gun at 1 MPa. A sheet of paper was placed in front of the filter to serve as a visual indicator of gas penetration through the filter. Changes in PM captured filters were imaged using SEM.

Omnidirectional filtering performance was evaluated under artificially generated random airflow. The filtering apparatus was fabricated by 3D printing a gyroid structure (unit cell size: 8 mm) using polylactic acid (PLA) with 0.2 mm of layer height, with an outer diameter of 8 cm and a height of 12 cm. A thin DIBA layer (∼500 nm) was fabricated on the gyroid structure by spray‐coating approximately 140 mg. The filtering apparatus was placed inside an acrylic chamber (30 × 30 × 30 cm). Eight fans were mounted on the four side walls to generate random airflow. A2 dust was continuously supplied into the chamber using a particle generator (SAG 410). Captured PMs on the apparatus after 6 h of filtration were demonstrated using a camera (EOS 80D) and SEM (SNE‐4500 M Plus).

### 4.5 Absorption Analysis

Absorption behavior was observed over polymer films coated on cover glass (Marienfeld). DIBA and polydimethylsiloxane (PDMS, Sylgard 184, Dow Corning) films were prepared by drop casting precursor solution onto the substrate. We used silica particles with diameters of 296.8 ± 21.3 and 1,215.4 ± 85.2 µm (Daehan Scientific), and polyethylene particles with diameter of 289.5 ± 22.5 µm (Cospheric). Fluorinated silica particles were prepared using 1H,1H,2H,2H‐perfluorooctyltriethoxysilane (98%, Sigma‐Aldrich). The particles were immersed in a 0.1 wt.% 1H,1H,2H,2H‐perfluorooctyltriethoxysilane solution in hexane (99% Samchun Chemicals), stirred for 1 h 30 min, and subsequently washed three times with hexane.

The absorption behavior was recorded using a MicroHD camera (Eakins). Particles were manually placed on the film surface. Absorption depth of the particle was quantified using MATLAB. Vertical intensity profiles were summed along the y‐direction, and the coordinate showing a sharp change in intensity was defined as the top surface of the particle. The absorption depth was calculated from the difference between the initial particle height upon contact and the final height after absorption. Lines following the particle surface was reconstructed using a circular profile derived from the particle position. Meniscus geometry was then manually measured along this reconstructed outline using ImageJ.

Absorption capacity of filter media was also evaluated. Polyester filters were coated with DIBA at loading of 97.0 ± 2.1 g m^−2^ and 13.4 ± 1.1 g m^−2^, and silicone oil (1,000 cSt, Gelest) was coated at 101.7 ± 2.0 and 14.2 ± 1.6 g m^−2^. N_2_ gas was then blown onto the coated filters using an air gun at 2 MPa, and the amount of removed coating layer was measured to evaluate the stability of applied layer. A2 dust was applied to the filter, and the dust‐captured filters were left for 24 h to allow the adhesive layer to absorb the particles. Subsequently, particles that were not absorbed by the adhesive layer were removed using an air gun. The absorption capacity was quantified by comparing the filter initial weight and final weight after dust absorption. The absorption capacity of DIBA for various particle type was also evaluated using planar substrates coated with DIBA films. Silica (5‐50 µm, Sigma‐Aldrich), polystyrene (9‐11 µm, MICROBEADS), poly(methyl methacrylate) (9–11 µm, MICROBEADS), and iron particles (Samchun) were deposited onto DIBA films. Unabsorbed particles were removed with air gun, and the absorption capacity was measured using an analytical balance. Morphology of absorbed particle within the polymer network was visualized by cutting the polymer film and imaging the cross‐section with SEM (SNE‐4500 M Plus).

### 4.6 Pressure Drop

Pressure drop during filtration was measured using a custom‐built air filter testing chamber. The experiments were designed to assess (1) the change in pressure drop induced by DIBA coating on high‐efficiency (HE) filter media, and (2) the operational lifetime of the filter. Polypropylene HE‐filters and polyester pre‐filter were employed with fiber diameters of 1.3 ± 0.4 and 33.1 ± 7.1 µm, respectively. To evaluate the effect of particle absorption on pressure drop, we monitored pressure drop during PM filtration and the subsequent resting period without further PM generation. Changes in pore area were analyzed using metal meshes to assess how the pores changed after filtering and subsequent absorption (Figure S24). Both bare and DIBA‐coated metal mesh were loaded with approximately 80 g m^−2^ of A2 dust. The pore area of bare meshes and DIBA‐coated meshes was observed using an optical microscope (S39B, Microscopes). Projected pore areas were quantified using ImageJ.

Additionally, to assess the operational lifetime of the filter, the time required for the pressure drop to reach twice its initial value was measured. In the multistage filtration system, the pre‐filter was positioned at the upstream of the HE‐filter. The screening effect of the pre‐filter was evaluated by comparing the pre‐filter and HE‐filter after PM filtration. The lifetime of bare pre‐filter with bare HE‐filter was used as the reference time for the DIBA pre‐filter with bare HE‐filter.

### 4.7 Computational Methods

We employed nonequilibrium molecular dynamics (NEMD) simulations to investigate the characteristics of stress relaxation and dynamic exchange reaction in the DIBA systems. The initial model was constructed in a three‐dimensional periodic simulation box containing 20 chains of aminopropylmethylsiloxane‐dimethylsiloxane copolymers (∼5,000 g mol^−1^) and 15 molecules of terephthalaldehyde. After energy minimization and enough equilibration, the crosslinking procedure between amine and aldehyde groups was implemented to generate an imine‐crosslinked network, followed by additional *NPT* and *NVT* simulations for ∼20 ns at 300 K and 1 atm (corresponding density is ∼1.01 g cm^−3^). To mimic the experimental drying condition, the number of water molecules in the simulation cell was systematically varied to construct four systems (crosslinking% = 50, 90, and 100%). Here, the degree of crosslinking was calculated from the consumption ratio of aldehyde groups, and the fraction of formed imine bonds was determined from the density functional theory (DFT)‐derived probability of dynamic bond exchange reaction. All simulations were performed using the large‐scale atomic/molecular massively parallel simulation (LAMMPS) [67] with the polymer consistent force field (PCFF) [68]. After equilibration, the stress relaxational behavior of each system was evaluated after imposing a 1% strain. Non‐bonded interactions and electrostatics were described using Lennard‐Jones (LJ) 12–6 potential and Ewald summation, respectively [69]. The visualizations from MD calculation were represented using Materials Studio 2025. To parameterize the probability of dynamic bond exchange, we carried out DFT calculations on individual molecules (aldehyde, amine, imine, and water) using Vienna ab initio Simulation Package (VASP) [70], with the Perdew‐Burke‐Ernzerhof (PBE) [71, 72] exchange‐correlation functional, D3 dispersion correction, and an implicit solvation effect. Based on the previous crosslinked aldehyde study [73], the free energies for imine formation and hydrolysis were converted into equilibrium constant via $K=\exp\;(-\Delta G^{\circ }R^{-1}T^{-1})$, which were then used to obtain the bond‐exchange probabilities in the NEMD simulations.

## Conflicts of Interest

The authors declare no conflicts of interest.

## Supporting information




**Supporting File 1**: adma73164‐sup‐0001‐SuppMat.docx.

**Supporting File 2**: adma73164‐sup‐0002‐MovieS1.mp4.

**Supporting File 3**: adma73164‐sup‐0003‐MovieS2.mp4.

**Supporting File 4**: adma73164‐sup‐0004‐MovieS3.mp4.

**Supporting File 5**: adma73164‐sup‐0005‐MovieS4.mp4.

## Data Availability

The data that support the findings of this study are available from the corresponding author upon reasonable request.
