# Supplementary material for: A Super‐Adhesive Air Filter With Capillarity‐Mediated Spontaneous Particle Absorption via Dynamic Bond Exchange
Source: Adv Mater. 2026 Apr 22;38(42):e00006. doi: 10.1002/adma.202600006 (PMC13410620; doi:10.1002/adma.202600006)
Supplement: Supplementary file 1 — Supporting File 1: adma73164‐sup‐0001‐SuppMat.docx. [file ADMA-38-e00006-s001.docx]

**A Super-Adhesive Air Filter with Capillarity-mediated Spontaneous Particle Absorption via Dynamic Bond Exchange**

Junyong Park^1^, Hyeri Jeon^1^, Hyunwoong Park^1^, Ji Min Lee^1^, Yeomyung Yoon^2^, Gyeong Hwan Choi^2^, Sazzadul A. Rahat^3^, Dong Wook Lee^4,5^, Sohdam Jeong^4,5^, Jonathan T. Pham^3,6^, Chae Bin Kim^2,7,8,9^* and Sanghyuk Wooh^1^*

*^1^Department of Chemical Engineering, Chung-Ang University, Seoul, 06974, Republic of Korea*

*^2^School of Chemical Engineering, Pusan National University, Busan, 46241, Republic of Korea*

*^3^Department of Mechanical and Materials Engineering, University of Cincinnati, Ohio, 45221, USA*

*^4^Department of Chemical Engineering, Dong-Eui University, Busan, 47340, Republic of Korea*

*^5^Center for Brain Busan 21 Plus Program, Dong-Eui University, Busan, 47340, Republic of Korea*

*^6^Department of Chemical and Environmental Engineering, University of Cincinnati, Ohio, 45221, USA*

*^7^Department of Polymer Science and Engineering, Pusan National University, Busan, 46241, Republic of Korea*

*^8^Research Institute of Industrial Technology, Pusan National University, Busan, 46241, Republic of Korea*

*^9^Research Institute for Convergence of Biomedical Science and Technology, Pusan National University Yangsan Hospital, Yangsan, 50612, Republic of Korea*

**Keywords**

: Particle adhesion, capillary force, dynamic bond polymer, air filter, viscoelasticity

*Corresponding authors. Email: [woohsh@cau.ac.kr](mailto:woohsh@cau.ac.kr), [cbkim@pusan.ac.kr](mailto:cbkim@pusan.ac.kr)


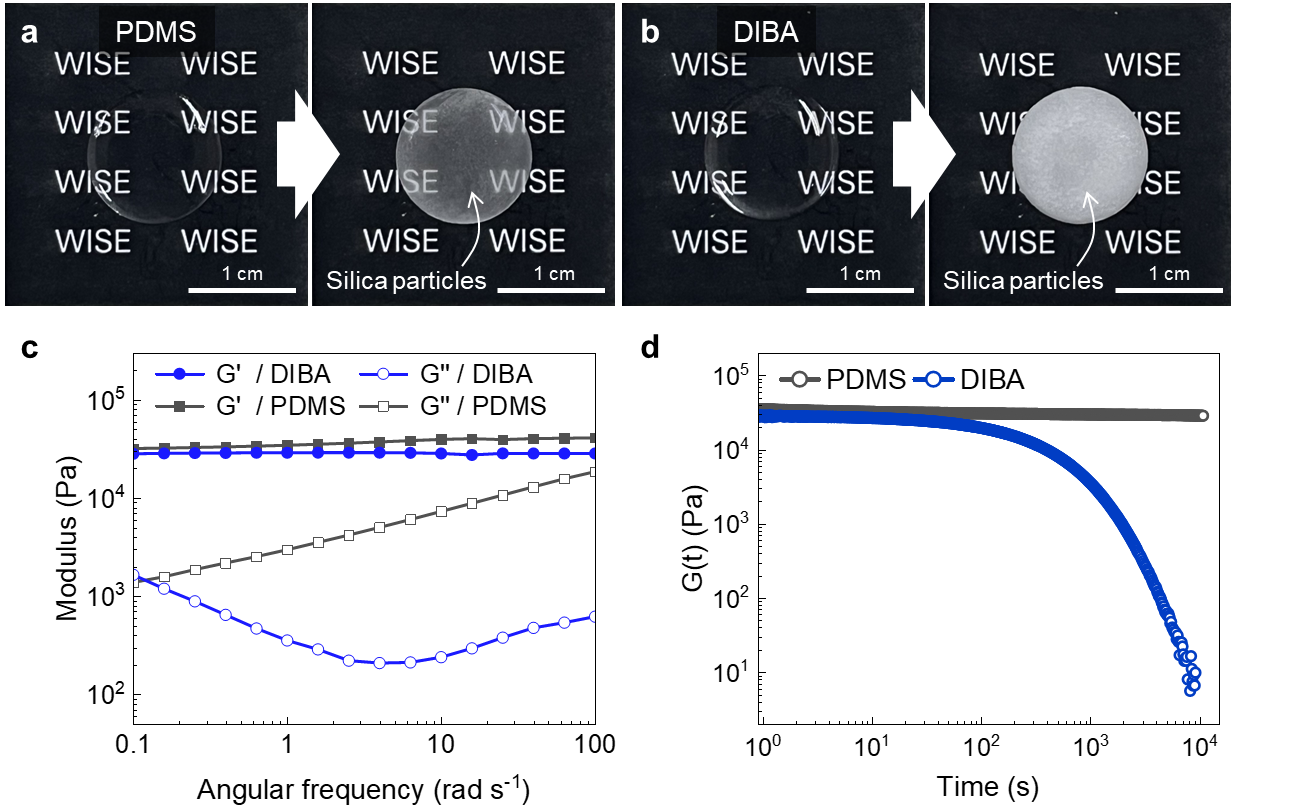


**Figure S1. Particle capturing and rheological properties by network type.** a, b) Photographic images of (a) permanently crosslinked polydimethylsiloxane (PDMS) and (b) dynamic imine bond adhesive (DIBA) coated glass substrate before and after silica particle deposition (5-50µm). c) Frequency sweep experiment of PDMS (gray) and DIBA (blue) at 1% strain and 25 ℃. d) Stress relaxation measurement of PDMS (gray) and DIBA (blue) at 1% strain and 25 ℃.


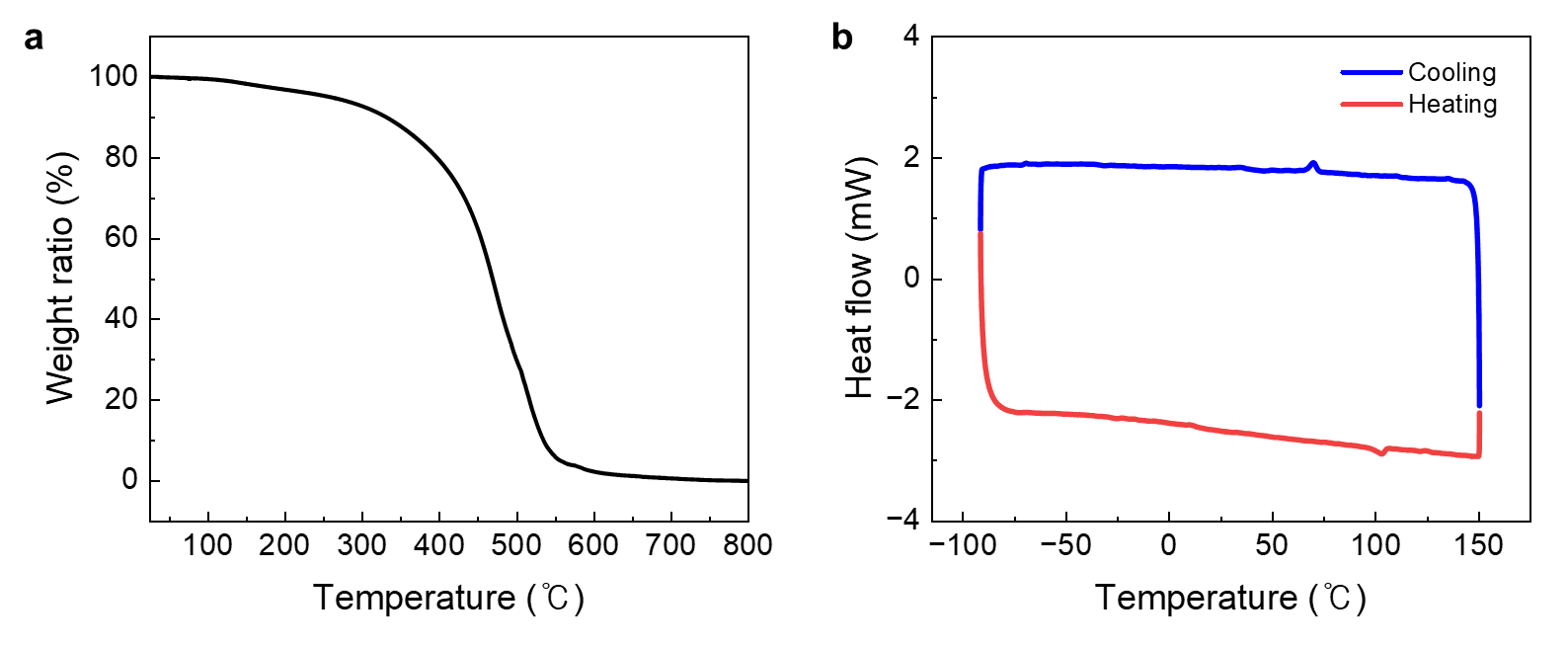


**Figure S2. Thermal characterization of the DIBA network.** a) Thermogravimetric analysis (TGA) of DIBA performed from 25 ℃ to 800 ℃ at a heating rate of 5 ℃ min^-1^ under nitrogen atmosphere. No significant mass loss is observed prior to thermal degradation above ~400 ℃, while a slight decrease above ~100 ℃ is attributed to the evaporation of matrix-confined water. b) Differential scanning calorimetry (DSC) curves of DIBA measured from -90 ℃ to 150 ℃ at a heating/cooling rate of 5 ℃ min^-1^ under nitrogen. The red and blue line corresponds to heating and cooling cycle, respectively.


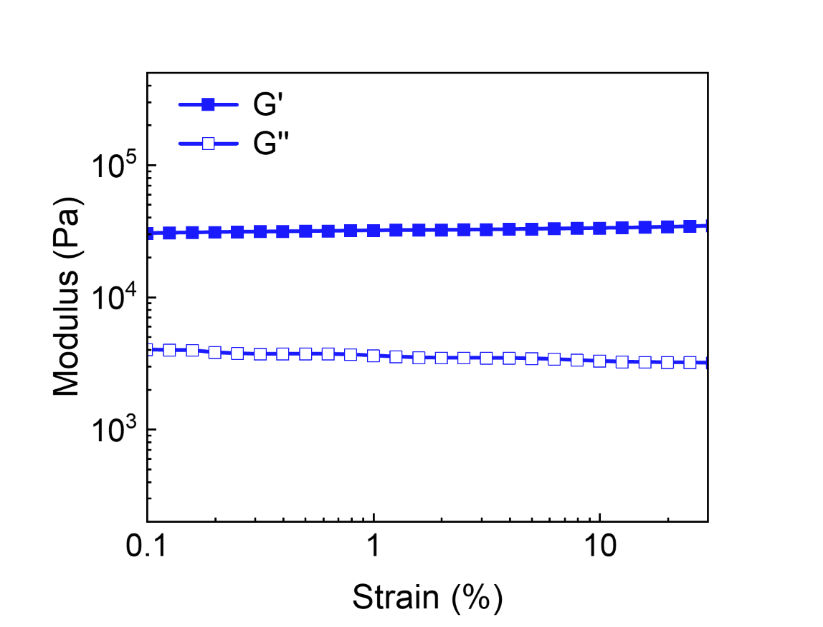


**Figure S3. Amplitude sweep experiment of DIBA.** All rheological experiments was operated under the linear viscoelastic region (LVR) as proven in amplitude sweep measurement. The DIBA mixed for 1:0.75 (prepolymer : crosslinker) in molar ratio was utilized at angular frequency of 1 rad s^-1^ and 25 ℃.


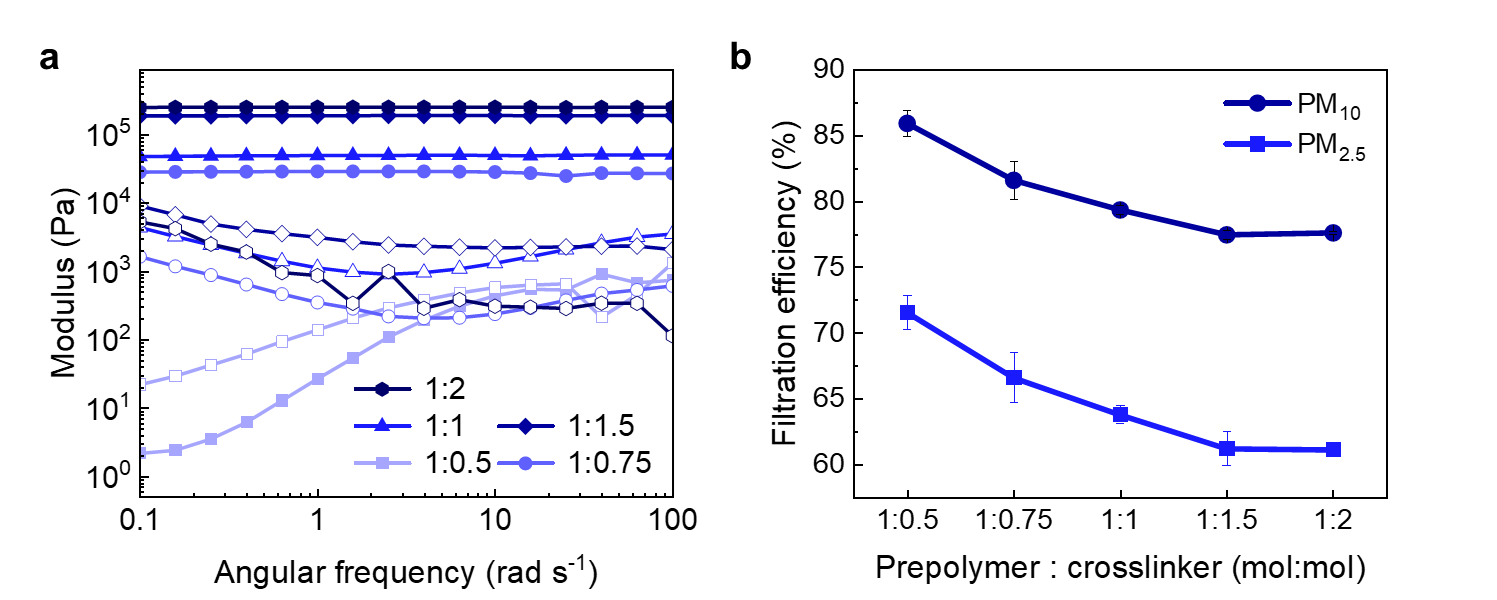


**Figure S4. Crosslinker ratio effect.** a) Frequency sweep curves as function of a mixing ratio of prepolymer (amine-modified PDMS) and crosslinker (terephthalaldehyde) at 1% strain and 25 ℃. Filled and open symbol represent the storage modulus (G’) and loss modulus (G’’), respectively. b) Filtering performance of DIBA polyester filter with varied mixing ratio. Dark navy, and blue line indicate filtration efficiency of PM_10_ and PM_2.5_, respectively.


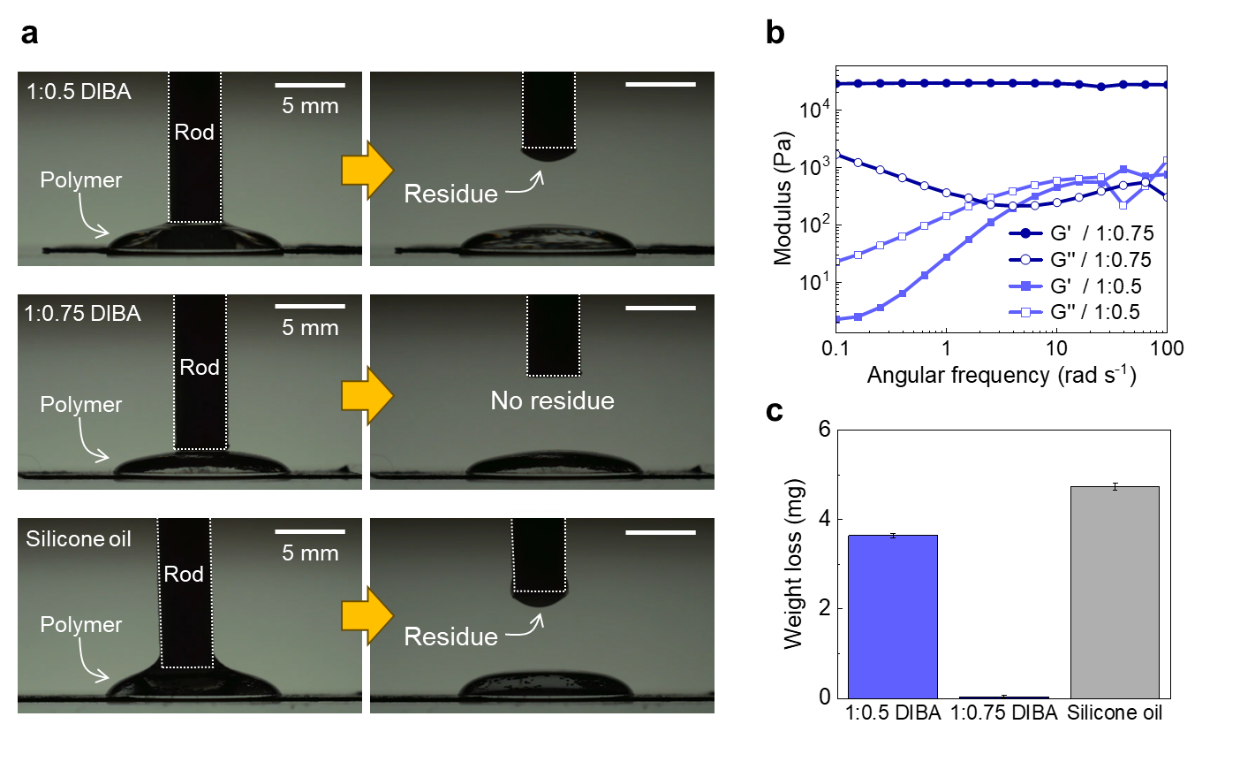


**Figure S5. Mechanical stability.** a) Images of rod interacting with polymer: 1:0.5 DIBA, 1:0.75 DIBA, and silicone oil 1,000 cSt. b) Frequency sweeps of 1:0.5 and 1:0.75 DIBA at 1% strain and 25 ℃. c) Weight loss of coated materials after mechanically interacting with rod. Its weight was measured using loadcell attached to rod.


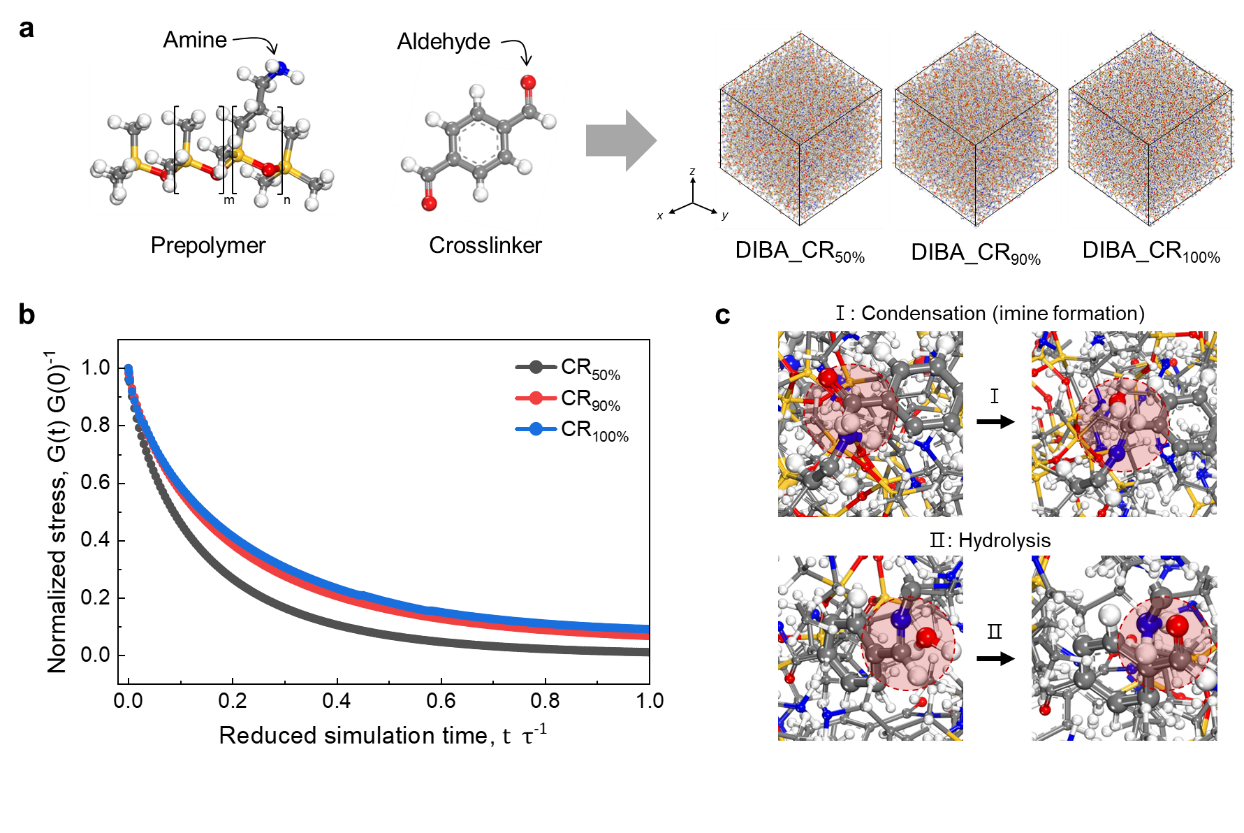


**Figure S6. Molecular dynamic simulation.** a) Molecular structures of the aminopropylmethylsiloxane-dimethylsiloxane copolymer (m = 59, n = 4) and terephthalaldehyde linker, along with the three simulated DIBA networks with different crosslinking% (50, 90, and 100%), defined by the extent of aldehyde group consumption. Here silicon, carbon, nitrogen, oxygen, and hydrogen atoms are shown in yellow, gray, blue, red, and white, respectively. b) Normalized stress relaxation curves of DIBA systems as a function of reduced simulation time ($t \tau^{-1}$). c) Representative atomistic snapshots of condensation (top) and hydrolysis (bottom) reactions in the DIBA networks. For clarity, the surrounding polymer matrix is illustrated with reduced atom and bond size, while reactive functional groups are emphasized.


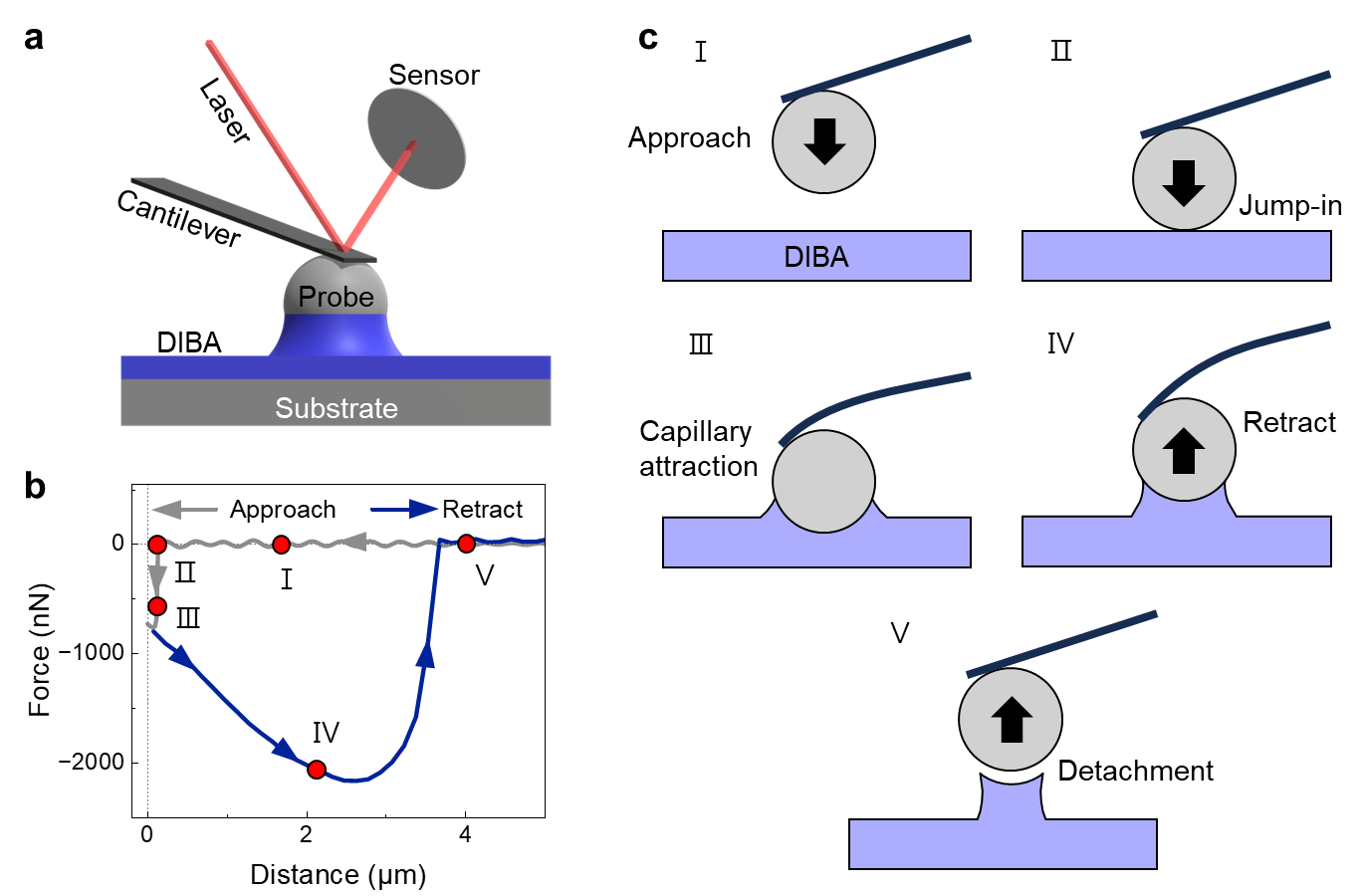


**Figure S7. Principles of force-distance curve.** a) Schematic of colloidal microprobe atomic force microscopy (AFM). b) Representative force-distance curve of approximately 10 µm silica microparticle interacting with 1:0.75 DIBA as depicted in Figure 3c. c) Schematic sequence of microparticle interacting with DIBA: Ⅰ. approach, Ⅱ. jump-in, Ⅲ. capillary attraction, Ⅳ. retract, Ⅴ. detachment.


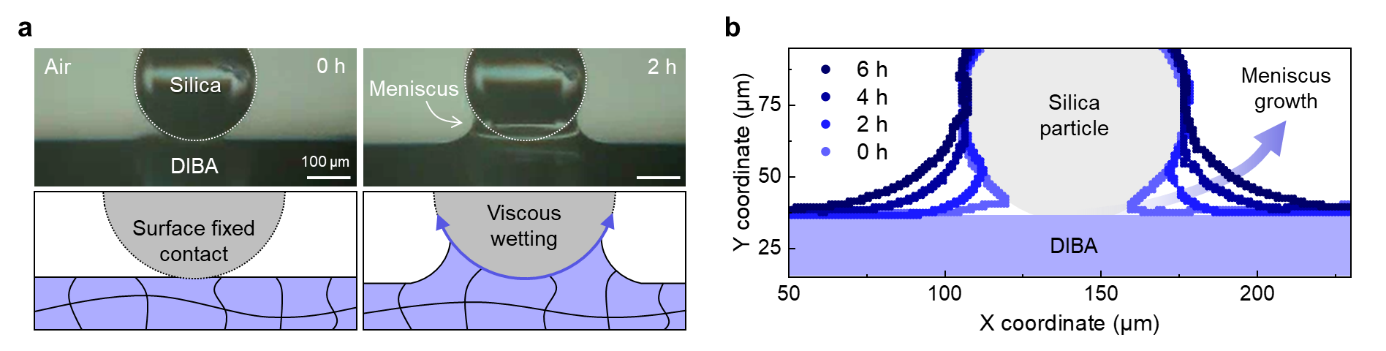


**Figure S8. Meniscus evolution of DIBA.** a) Snapshots (top) and schematics (bottom) demonstrating the wetting behavior of DIBA on a silica microparticle (≈ 300 µm). Wavy segment in black indicates crosslinked network of DIBA. b) Meniscus profile of DIBA as a function of surface contact time. Silica particle only contact at the surface of DIBA that was confirmed through changes in loadcell.


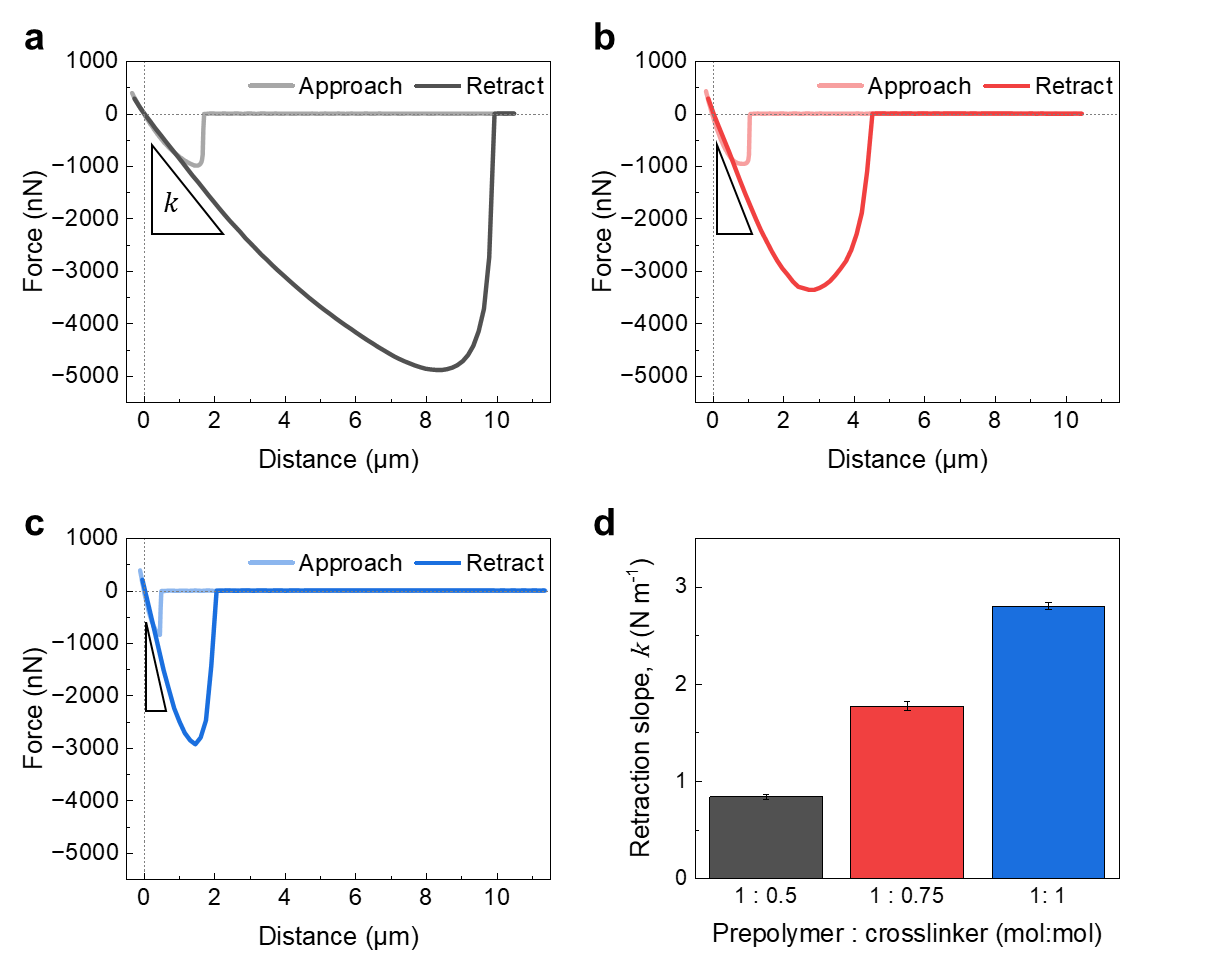


**Figure S9. Force-distance curve as function of a crosslinking ratio.** a-c) Force-distance curve of (a) 1:0.5, (b) 1:0.75, and (c) 1:1 DIBA. Silica microparticle (10 µm) was brought into contact with DIBA. An indentation force of 500 nN was applied. d) Retraction slope $k$ by crosslinking ratio. Slope was calculated particle retracting for 500 nm.


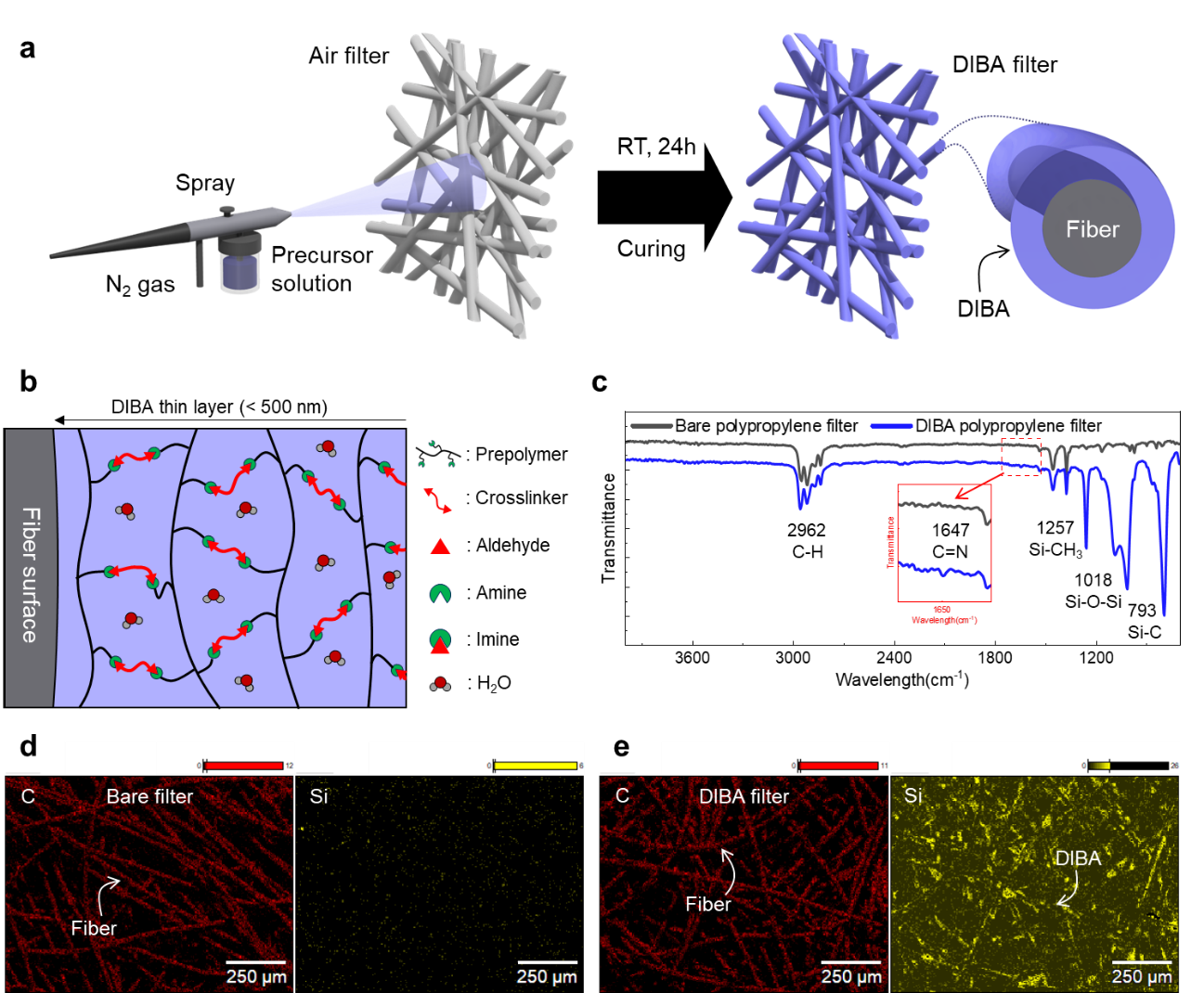


**Figure S10. Fabrication of DIBA coated filter.** a) Schematic of spray coating a precursor solution over pristine filter media and DIBA layer over filter fiber after curing under room temperature for 24 h. b) Schematic of crosslinked DIBA over filter fiber. c) Fourier-transform infrared (FTIR) spectroscopy of bare (gray) and DIBA (blue) coated polypropylene filter. d, e) Energy dispersive X-ray spectroscopy (EDS) images of (d) bare and (e) DIBA coated filter.


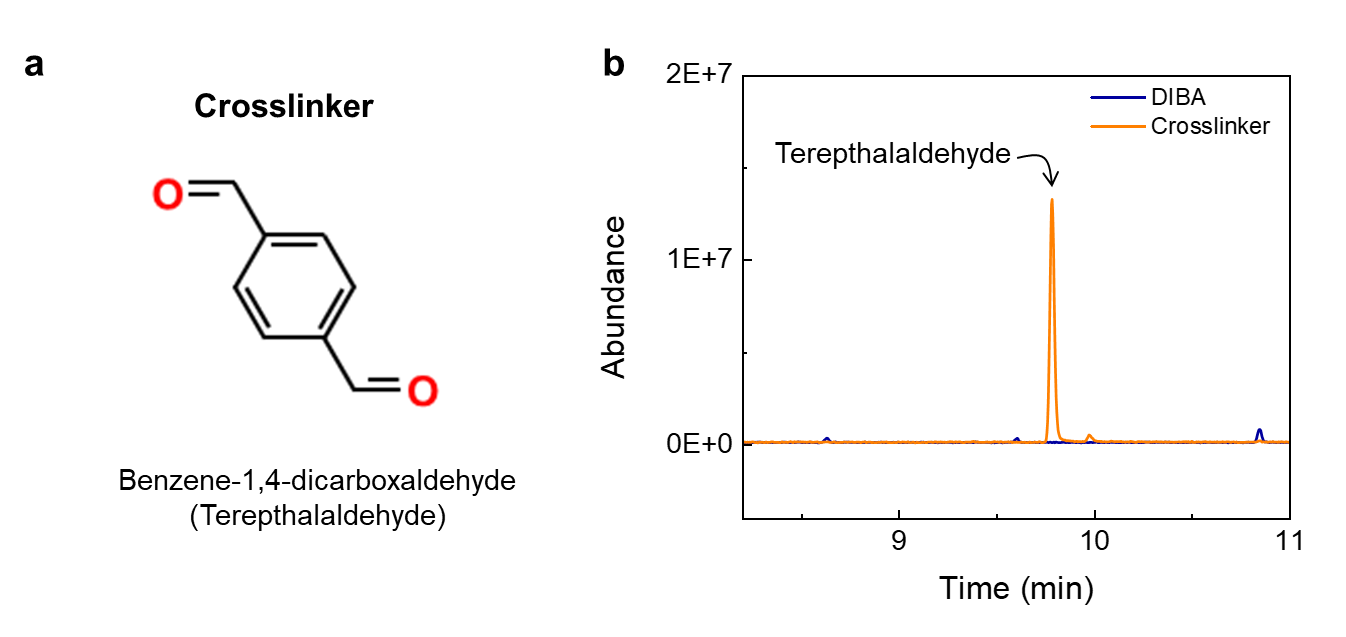


**Figure S11. Gas chromatography-mass spectrometry (GC-MS) analysis of DIBA and the crosslinker.** a) Chemical structure of crosslinker (i.e., terephthalaldehyde) of DIBA. b) Chromatograms of DIBA (blue) and crosslinker (orange). A distinct peak at approximately 9.7 min is observed for the crosslinker and assigned to terephthalaldehyde, whereas no corresponding peak is detected in the DIBA sample.


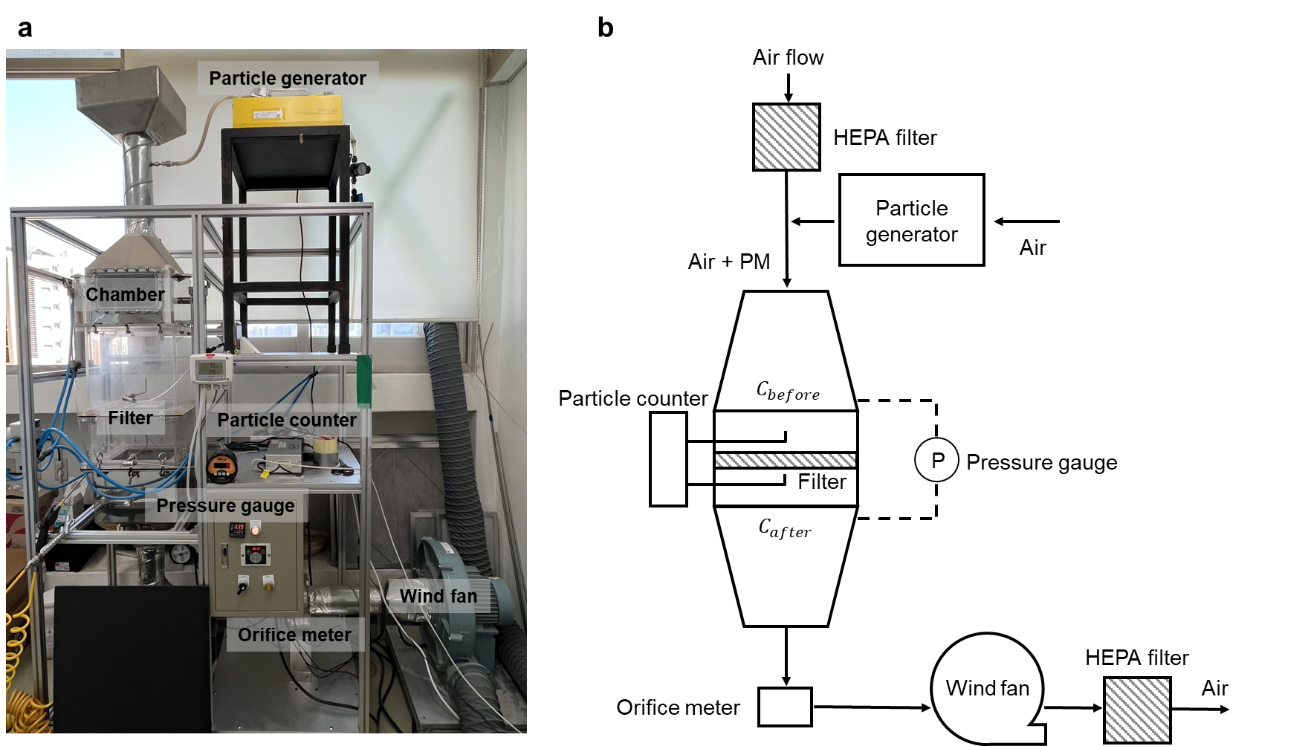


**Figure S12. Air filter testing chamber.** a, b) (a) Photographic image and (b) schematic design of air filter testing chamber following DIN 71460-1:2006 standard.

**Table S1. Filtering performances.** Detailed data shown in **Figure 4g**, including media type, air permeability at 125 Pa, filtration efficiency of PM_2.5_ (0.3-2.5 µm) and PM10 (2.5-10 µm) and pressure drop.


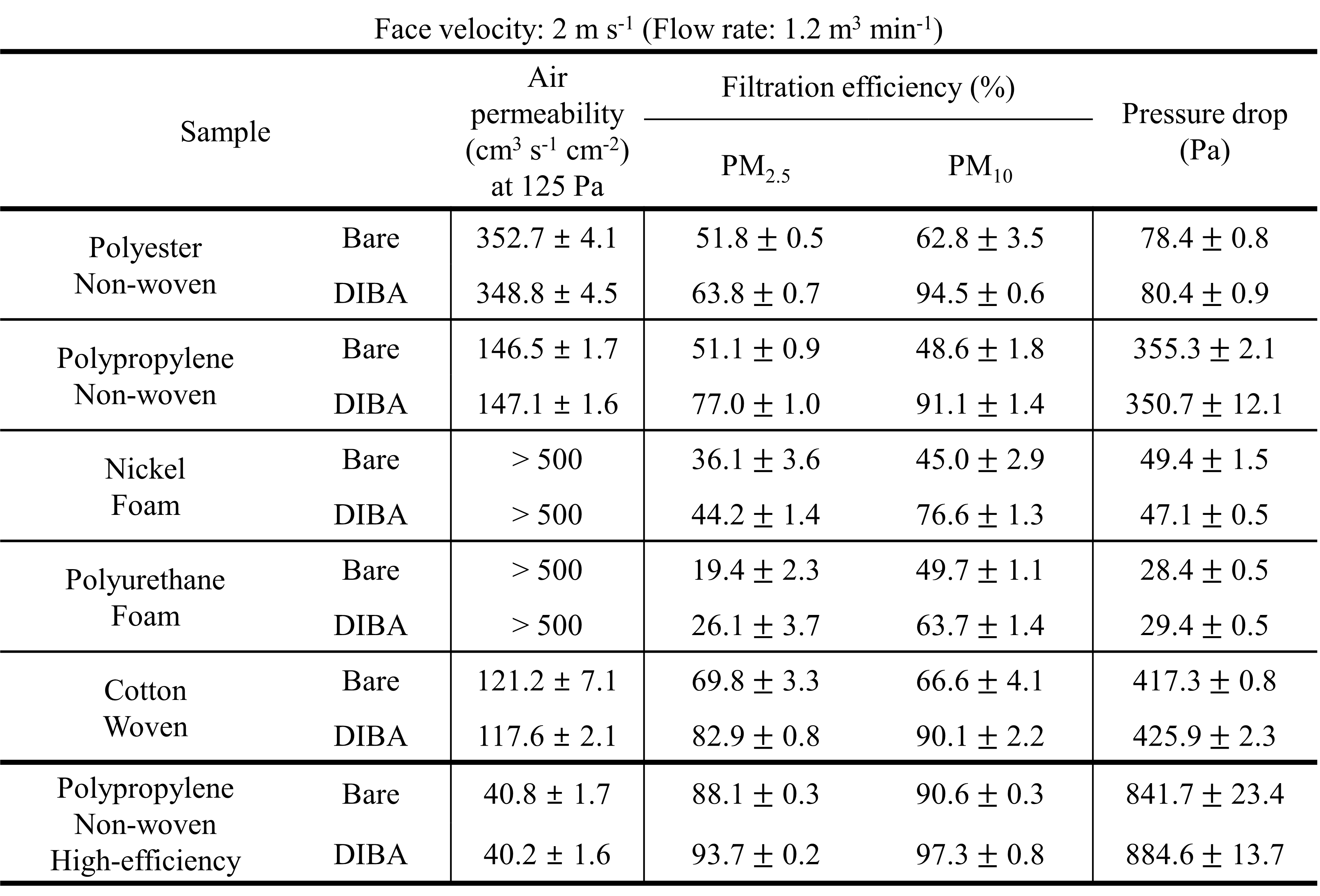


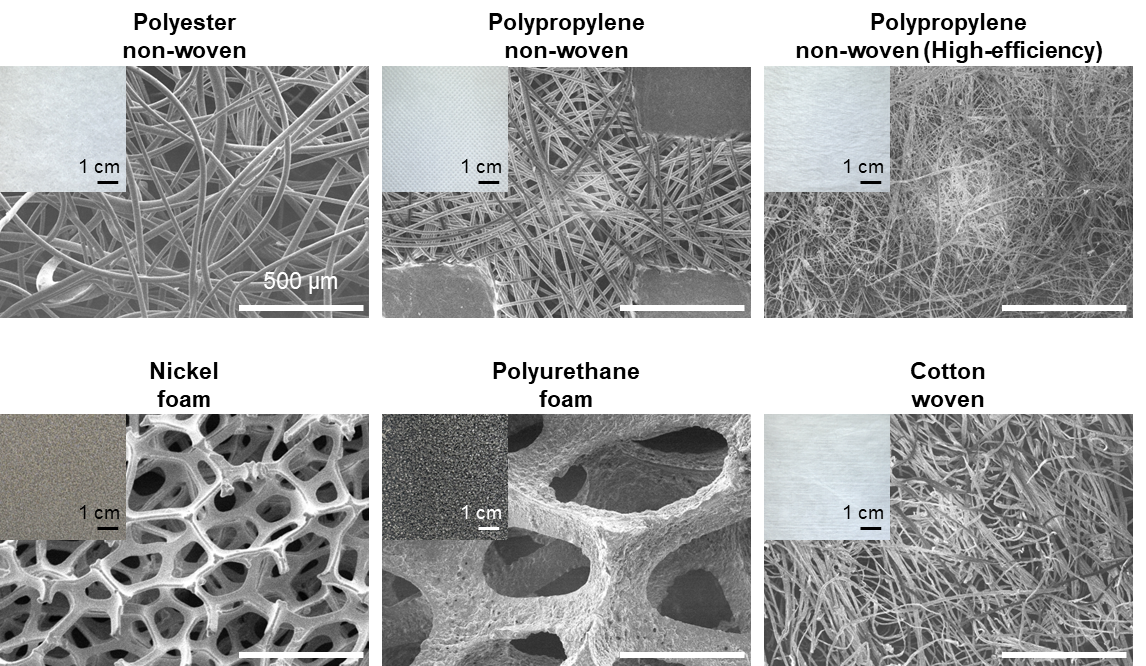


**Figure S13. Structure of filter medias.** Scanning electron microscope (SEM) and inset photographic images of polyester non-woven, polypropylene non-woven, high-efficiency polypropylene non-woven, nickel foam, polyurethane foam, and cotton woven filter media.


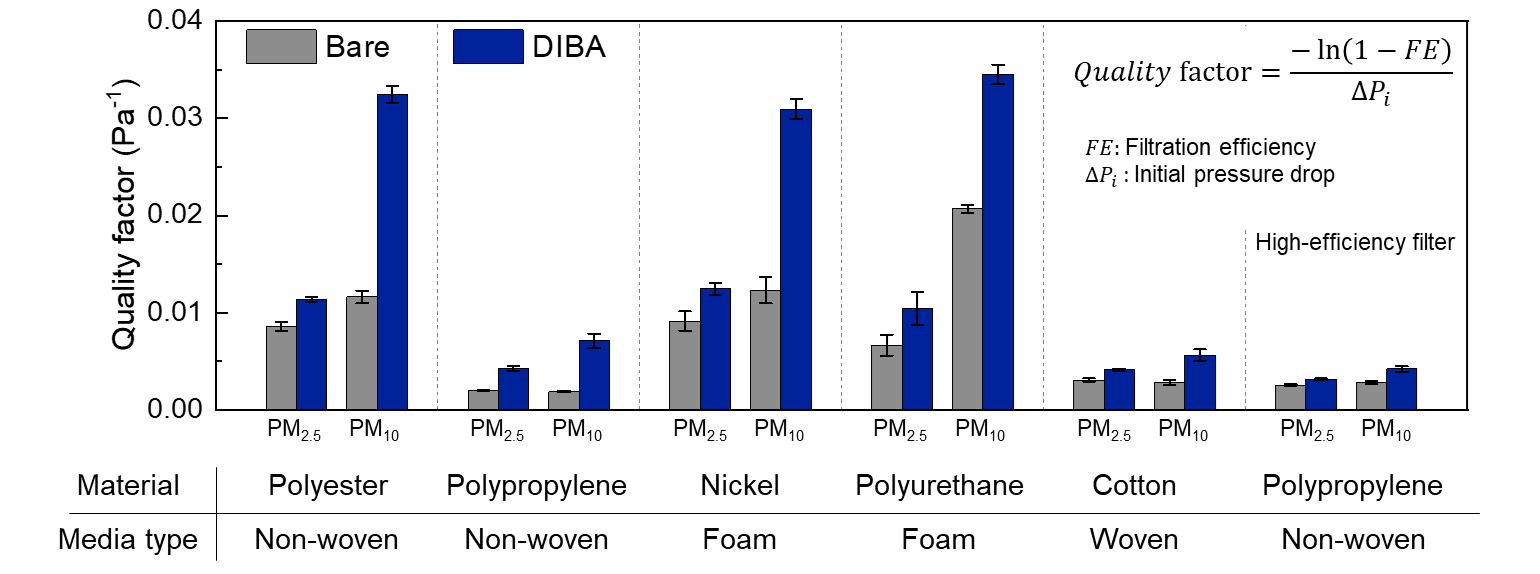


**Figure S14. Quality factor.** Quality factor (QF) of filters following the measured filtration efficiency at **Figure 4g**. Inset equation was used to calculate QF.


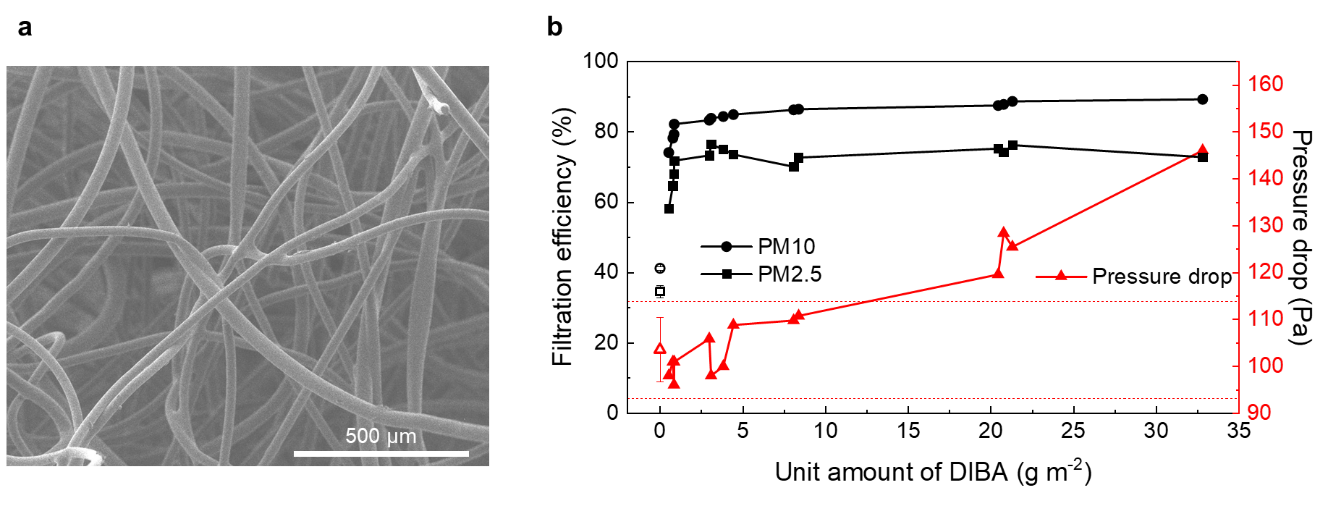


**Figure S15. Filtration performance with varied amount of DIBA.** a) SEM image of polyester filters with 380.2 ± 7.2 cm^3^ s^-1^ cm^-2^ of air permeability. b) Filtration performances of polyester filter coated between 0 g m^-2^ (i.e., uncoated filter) and 32.8 g m^-2^ of DIBA. Black and red lines demonstrating filtration efficiency and pressure drop of filter. Red dashed line indicate ± 10% deviation from the pressure drop of the uncoated filter, defining the acceptable operating range; pressure drop exceeding this range are considered to reflect coating-induced pressure increase.


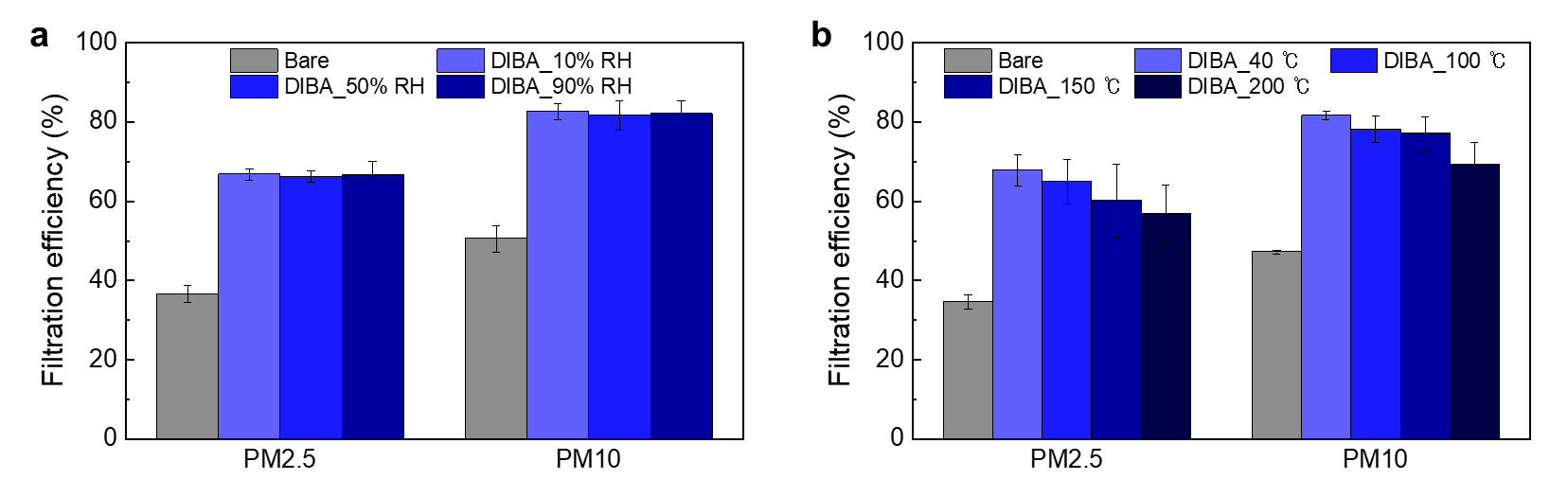


**Figure S16. Filtration performance of DIBA filter after environmental conditioning.** The DIBA network was prepared using a base to crosslinker molar ratio of 1:0.75. a) Filtration efficiency of polyester filter after storage at relative humidity (RH) levels (10%, 50%, and 90% RH) for 24 h; humidity conditioning was performed following drying at 40 ℃ as the baseline state. b) Filtration efficiency of polyester filter measured upon thermal conditioning at 40, 100, 150, and 200 ℃ for 24 h prior to testing at room-temperature (25 ℃).


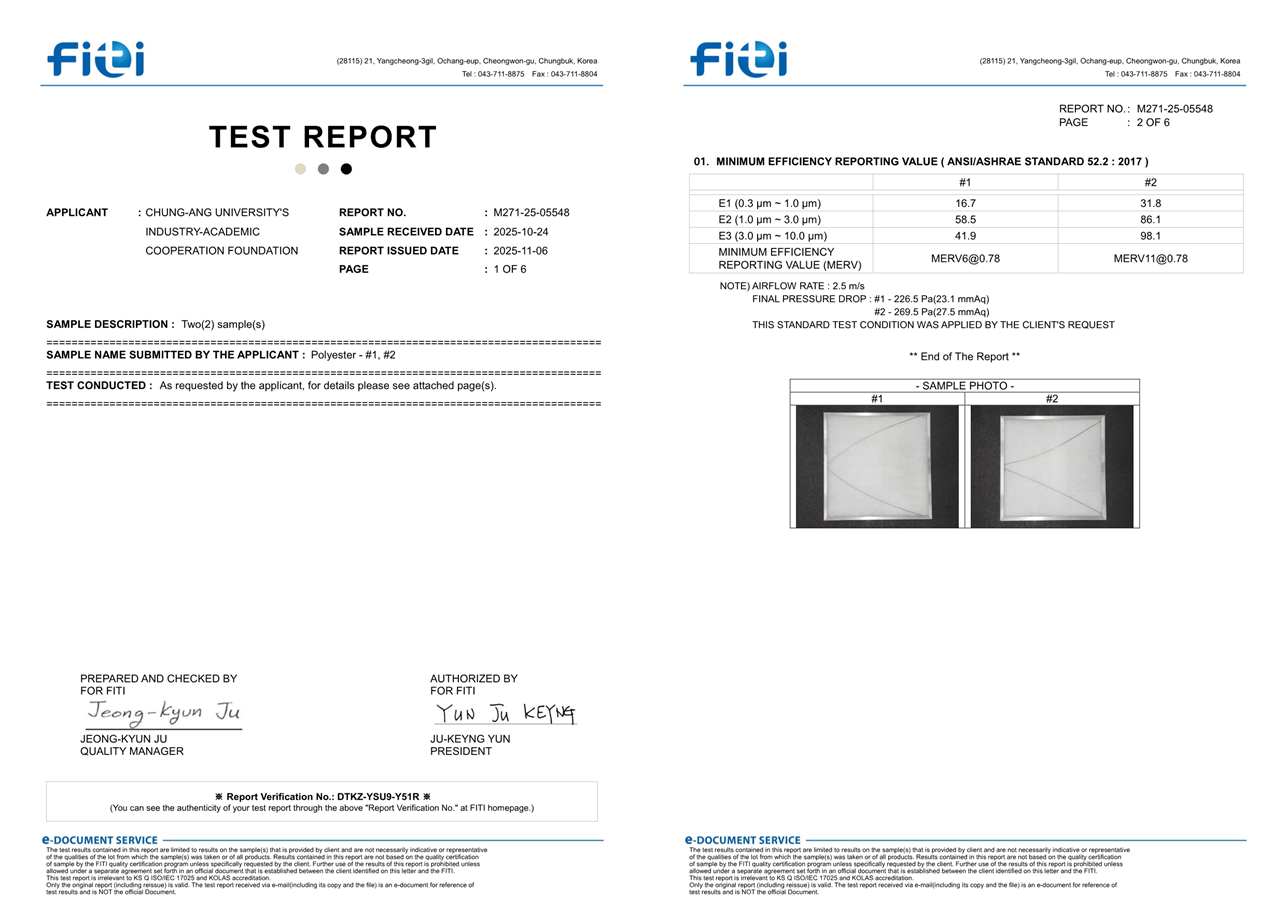


**Figure S17. Certificates of filtering performance.** Test report of bare polyester (#1) and DIBA polyester (#2) filter with dimension of 594 × 594 mm. Testing condition follows ANIS/ASHRAE Standard 52.2:2017 with 2.5 m s^-1^ of face velocity. The minimum efficiency reporting value (MERV) classification indicates industry standard for air filtration; the bare polyester filter corresponds to MERV 6 (pre-filter grade), whereas the identical polyester filter with DIBA coating achieves MERV 11 (medium-filter grade).


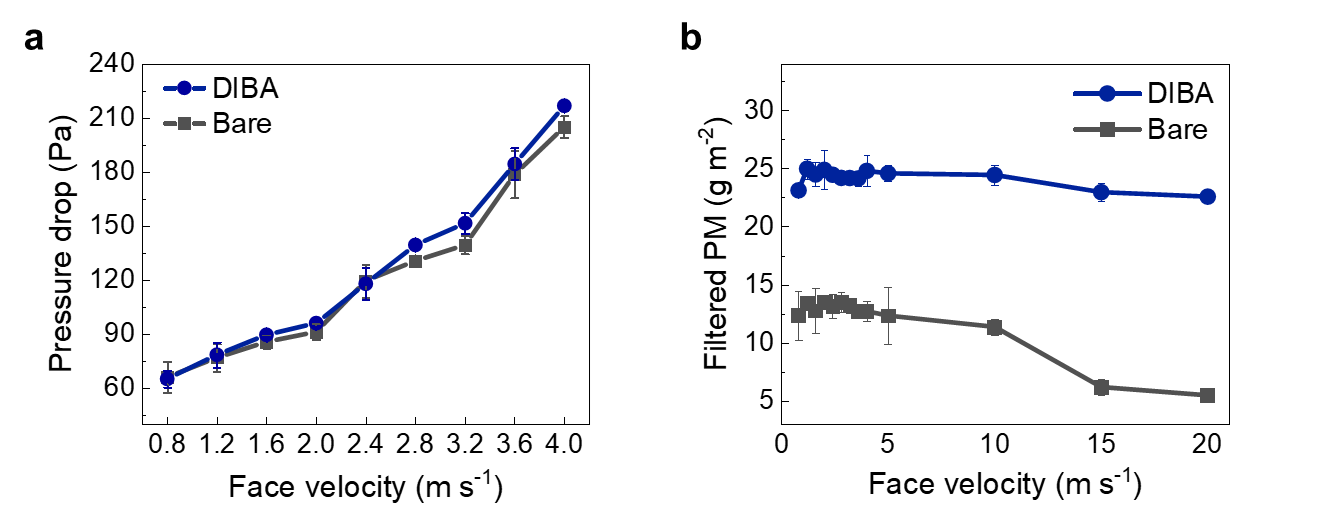


**Figure S18. Filtration performance with varied face velocity.** a) Pressure drop of bare (gray) and DIBA (blue) polyester filter as function of a face velocity. b) Filtered PM across face velocities from 0.8 m s^-1^ to 20 m s^-1^.


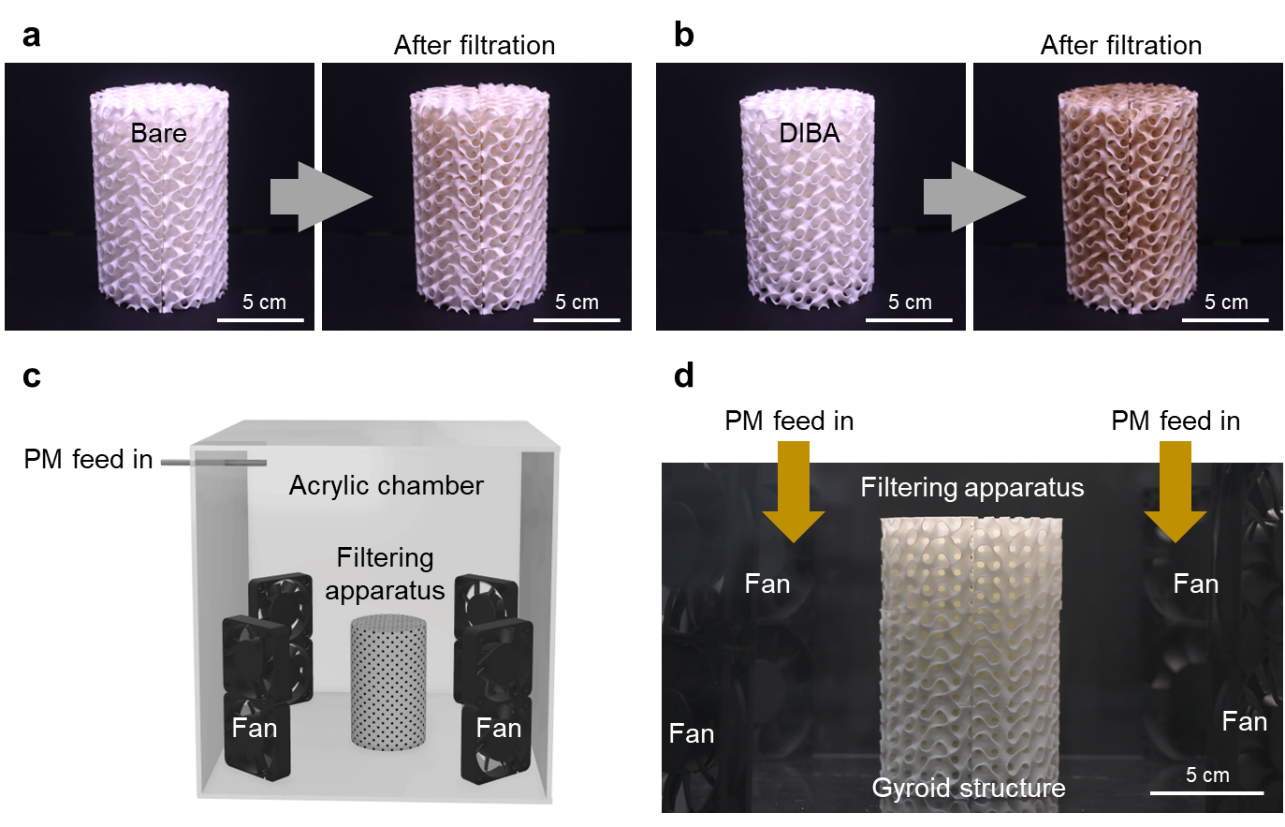


**Figure S19. Omnidirectional filtration.** a, b) Photographic images of (a) bare and (b) DIBA filtering apparatus before and after omnidirectional filtration. Gyroid structure was 3D-printed with 0.2 mm of layer height. c, d) (c) Schematic and (d) photographic overview of testing setup for omnidirectional filtration using acrylic chamber (300 × 300 × 300 mm). Filtering apparatus was placed in the middle of eight fans directed in four directions.


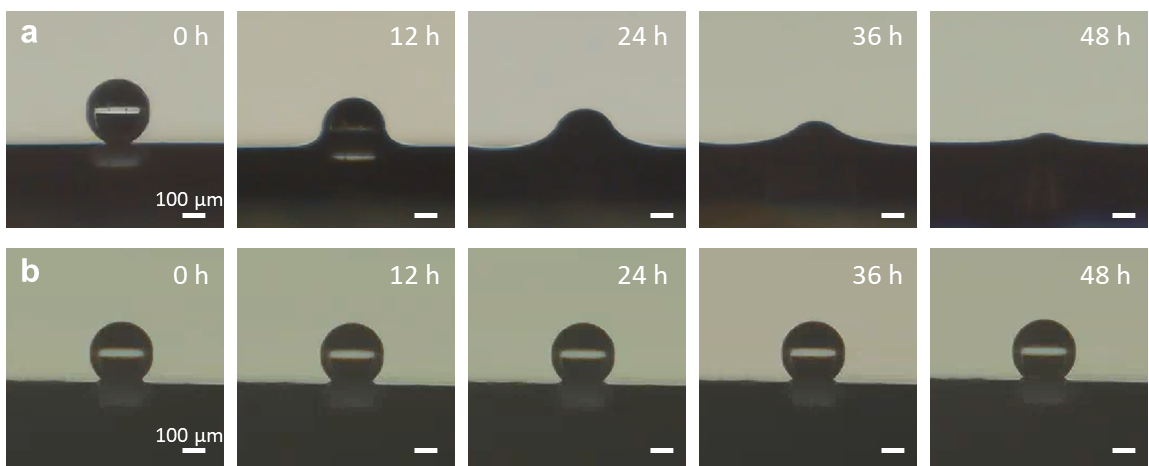


**Figure S20. Microparticle absorption by crosslinking type.** a, b) Snapshots of silica microparticle (≈ 300 µm) absorption of (a) dynamically crosslinked DIBA, and (b) permanently crosslinked PDMS matrix. Storage modulus (G’) of each polymer matrix was matched, as depicted in **Figure S1**.


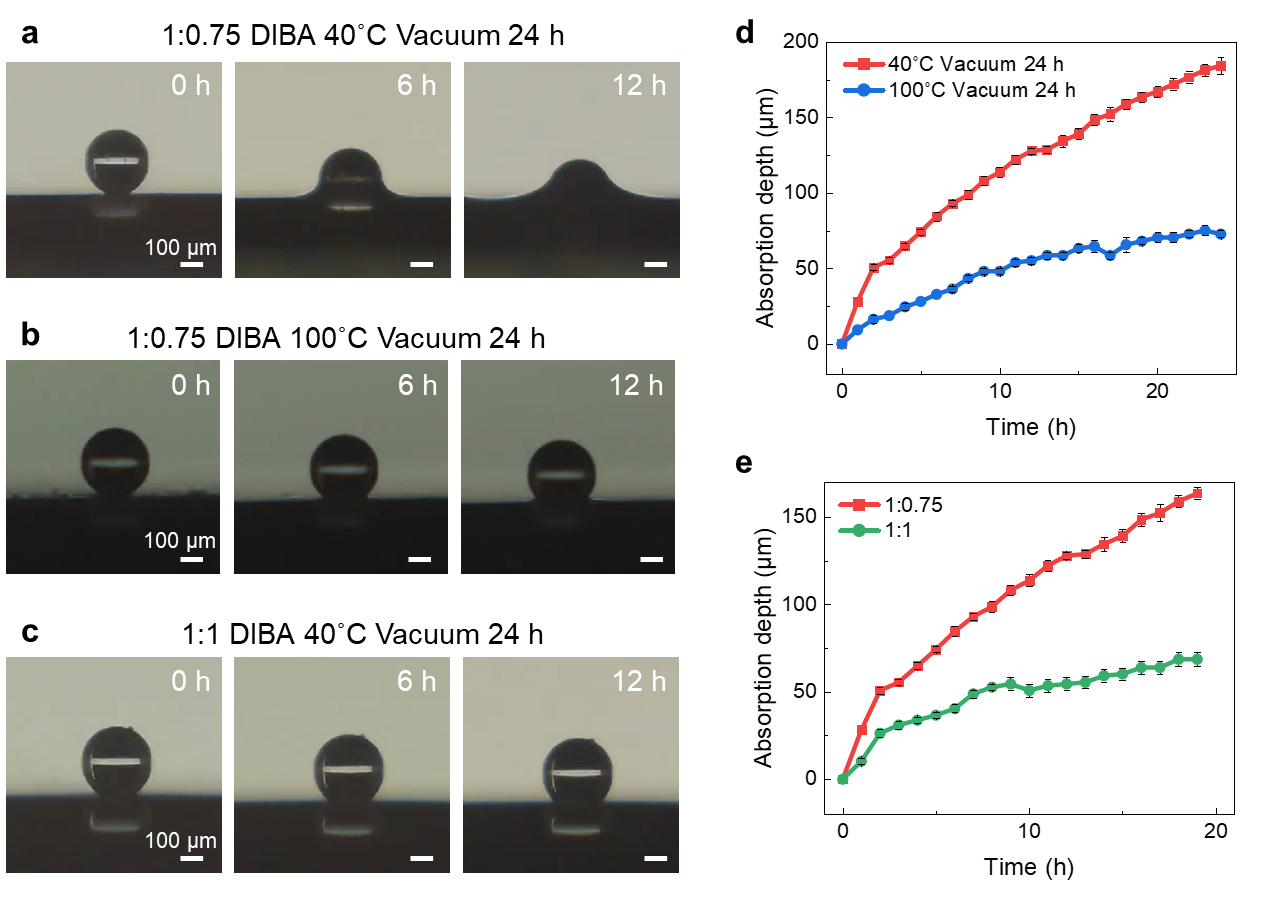


**Figure S21. Microparticle absorption by modulus.** a-c) Snapshots of 1:0.75 DIBA drying under (a) 40 ℃ vacuum 24 h, (b) 100 ℃ vacuum 24 h, and (c) 1:1 DIBA drying under 40 ℃ vacuum 24 h during absorption of silica microparticle (≈ 300 µm). d) Absorption depth of 1:0.75 DIBA drying under 40 ℃ vacuum 24 h (red) 100 ℃ vacuum 24 h (blue). e) Absorption depth of 1:0.75 (red) and 1:1 (green) DIBA drying under 40 ℃ vacuum 24 h.


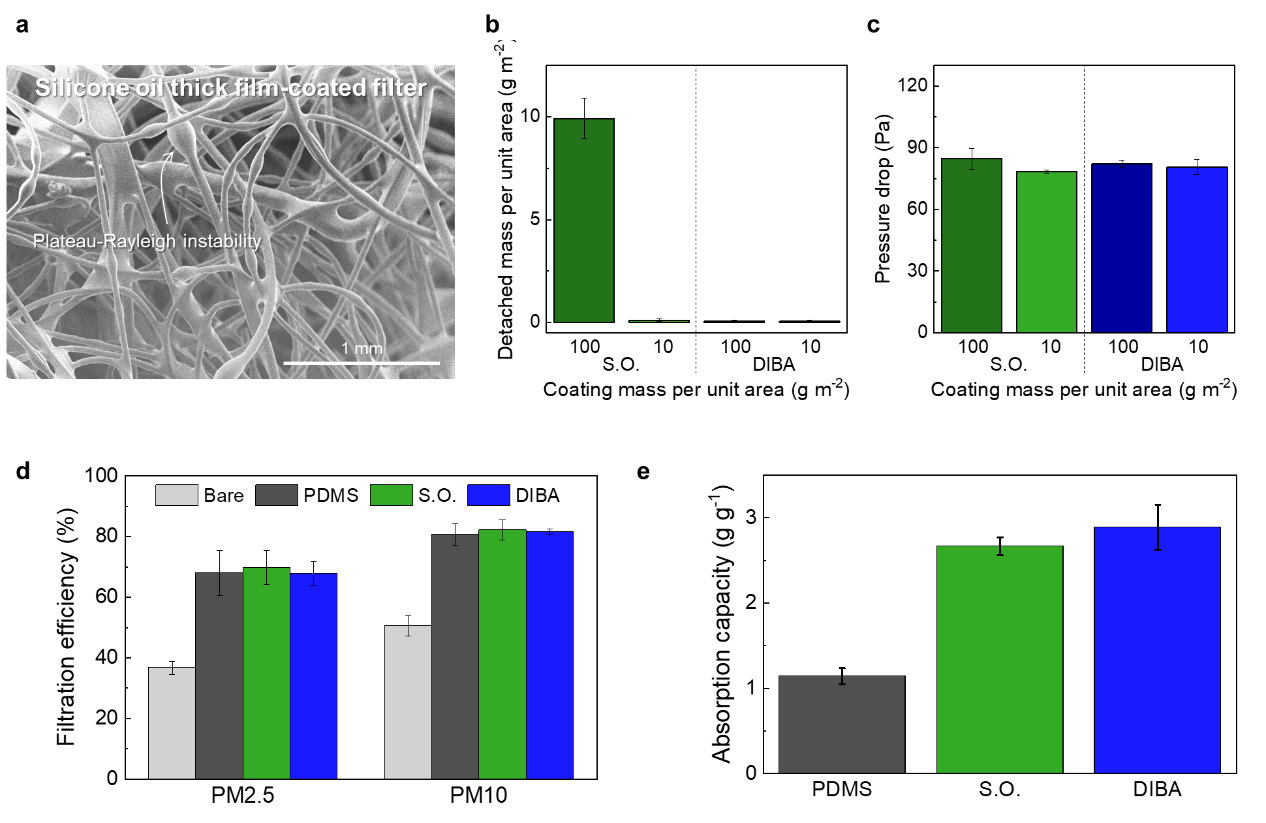


**Figure S22. Coating stability and absorption capacity with varied coating material.** a) SEM image of polyester filter coated with 100 g m^-2^ of silicone oil (i.e., S.O.). Due to think layer of liquid, Plateau-Rayleigh instability induced droplet was formed, indicated in white arrow. b) Detached mass per unit area of filter after blowing N_2_ gas. c) Initial pressure drop of S.O. (green) and DIBA (blue) coated polyester with thick (~100 g m^-2^) and thin film (~10 g m^-2^). d) Filtration efficiency of bare (gray), permanently crosslinked PDMS (dark gray), S.O. (green), and DIBA (blue) polyester filters. e) Absorption capacity of A2 dust per unit polymer mass for PDMS, S.O. and DIBA filters.


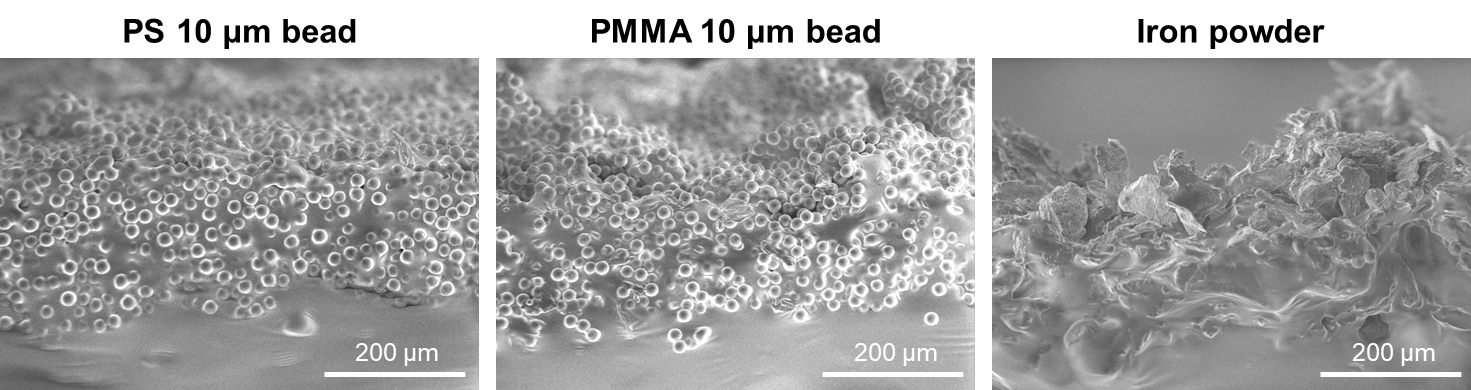


**Figure S23. Microparticle absorption into matrix.** SEM images of cross-section of DIBA after absorbing ~10 µm polystyrene (PS, left), ~10 µm poly(methyl methacrylate) (PMMA, middle), and iron powder (right).


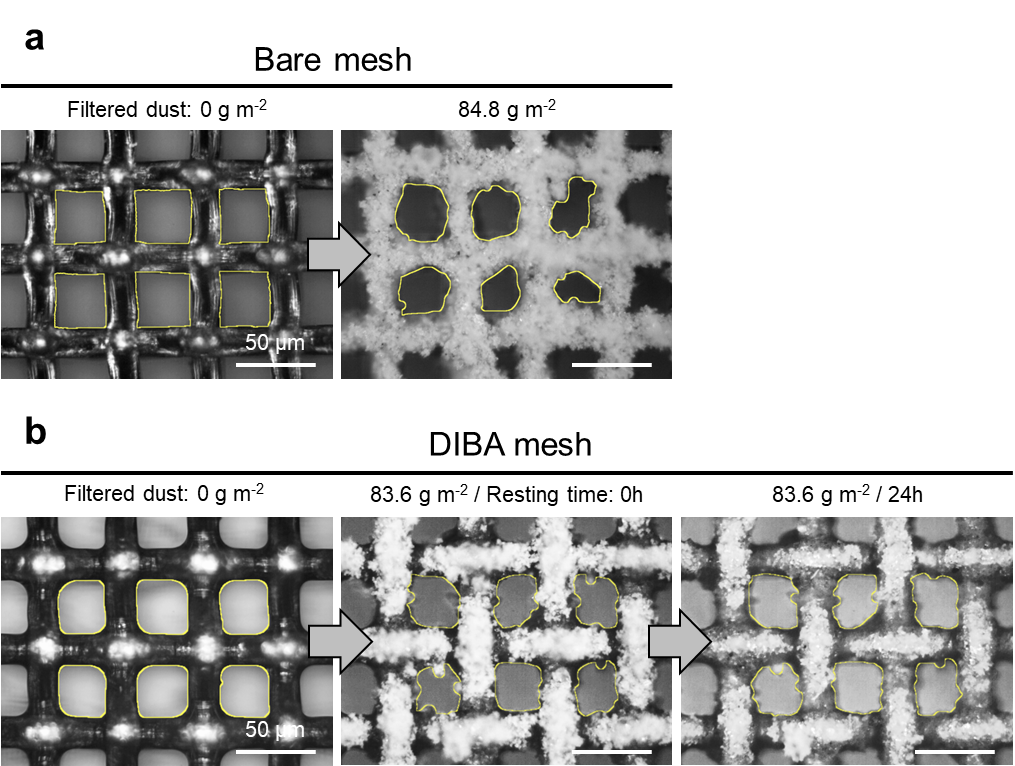


**Figure S24. Pore area changes in metal mesh.** a, b) Optical microscopic images of (a) bare and (b) DIBA-coated metal mesh before and after PM filtration. Each metal mesh (50 × 50 mm) captured approximately 80 g m^-2^ of PM. The particle absorption behavior of the DIBA-coated mesh was monitored for 24 h, as shown in **Movie S4**.
